# Single-cell spatial transcriptomics reveals distinct patterns of dysregulation in non-neuronal and neuronal cells induced by the *Trem2*^R47H^ Alzheimer’s risk gene mutation

**DOI:** 10.1038/s41380-024-02651-0

**Published:** 2024-08-05

**Authors:** Kevin G. Johnston, Bereket T. Berackey, Kristine M. Tran, Alon Gelber, Zhaoxia Yu, Grant R. MacGregor, Eran A. Mukamel, Zhiqun Tan, Kim N. Green, Xiangmin Xu

**Affiliations:** 1 Department of Anatomy and Neurobiology, School of Medicine, University of California, Irvine, CA 92697 USA; 2 Department of Biomedical Engineering, University of California, Irvine, CA 92697 USA; 3 Department of Neurobiology and Behavior, Charlie Dunlop School of Biological Sciences, University of California, Irvine, CA 92697 USA; 4 Department of Cognitive Science, University of California, San Diego, CA 92037 USA; 5 Department of Statistics, School of Computer and Information Science, University of California, Irvine, CA 92697 USA; 6 Center for Neural Circuit Mapping, University of California, Irvine, CA 92697 USA; 7 Department of Developmental and Cell Biology, University of California, Irvine, CA 92697 USA; 8 Institute for Memory Impairments and Neurological Disorders (UCI MIND), Irvine, USA; 9 Department of Molecular Biology and Biochemistry School of Biological Sciences, University of California, Irvine, CA 92697 USA

**Keywords:** Molecular biology, Neuroscience, Cell biology

## Abstract

The R47H missense mutation of the TREM2 gene is a known risk factor for development of Alzheimer’s Disease. In this study, we analyze the impact of the *Trem2*^R47H^ mutation on specific cell types in multiple cortical and subcortical brain regions in the context of wild-type and 5xFAD mouse background. We profile 19 mouse brain sections consisting of wild-type, *Trem2*^R47H^, 5xFAD and *Trem2*^R47H^; 5xFAD genotypes using MERFISH spatial transcriptomics, a technique that enables subcellular profiling of spatial gene expression. Spatial transcriptomics and neuropathology data are analyzed using our custom pipeline to identify plaque and *Trem2*^R47H^-induced transcriptomic dysregulation. We initially analyze cell type-specific transcriptomic alterations induced by plaque proximity. Next, we analyze spatial distributions of disease associated microglia and astrocytes, and how they vary between 5xFAD and *Trem2*^R47H^; 5xFAD mouse models. Finally, we analyze the impact of the *Trem2*^R47H^ mutation on neuronal transcriptomes. The *Trem2*^R47H^ mutation induces consistent upregulation of *Bdnf* and *Ntrk2* across many cortical excitatory neuron types, independent of amyloid pathology. Spatial investigation of genotype enriched subclusters identified spatially localized neuronal subpopulations reduced in 5xFAD and *Trem2*^R47H^; 5xFAD mice. Overall, our MERFISH spatial transcriptomics analysis identifies glial and neuronal transcriptomic alterations induced independently by 5xFAD and *Trem2*^R47H^ mutations, impacting inflammatory responses in microglia and astrocytes, and activity and BDNF signaling in neurons.

## Introduction

Genome-wide association studies (GWAS) have identified multiple genetic variants associated with Alzheimer’s Disease (AD) [1]. One key discovery identified in the TREM2 (Triggering receptor expressed on myeloid cell 2) gene is the R47H missense variant, a strong risk factor for development of Late-Onset Alzheimer’s Disease (LOAD) [2, 3]. TREM2 is an immunomodulatory cell surface receptor expressed primarily in microglia in the brain [4, 5], and is activated by a variety of ligands including amyloid-beta (Aβ), APOE, and phospholipids [6]. The R47 residue of TREM2 is located within a poly-basic region of the extracellular Ig-like domain, and may modify interactions of TREM2 with its associated ligands [7, 8].

In AD, microglia exhibit an inflammatory response to Aβ plaques both in human AD brains and in animal disease models [9, 10]. Evidence increasingly implicates regulation of microglia activation in several AD-related processes including plaque formation and growth [11], plaque compaction [11, 12], protection against dystrophic neurites [13], regulation of development and spread of Tau pathology [14], destruction of perineuronal nets [15, 16], and synaptic and neuronal loss [15, 17–20], though the role of microglia in suppressing or aggravating impacts of AD is currently unclear, and may vary with disease progression [21].

Recent efforts have produced a mouse model of the *Trem2*^R47H^ mutation in which bulk RNA-seq analysis has identified a unique *Trem2*^R47H^ induced interferon signature present at 12 months of age, believed to be associated with microglia in response to A*β* pathology [22]. Activation of microglia significantly impacts neuronal function and can be neurotoxic [23], but resolving the cell type-specific impacts of this mutation on neuronal populations requires single-cell analysis. Additionally, proximity to A*β* plaques directly impacts glial activation and neuronal transcriptomes [24], requiring spatial transcriptomics to analyze the combined influence of these effects.

To analyze the impacts of the *Trem2*^R47H^ mutation in the context of plaque pathology, we utilized the 5xFAD mouse model, noted for exhibiting strong Aβ pathology at relatively early ages [25]. The 5xFAD mouse model has been comprehensively evaluated for preclinical testing applications [26, 27]. It has been shown that different brain regions (i.e. cortex and hippocampus) in the 5xFAD model have both common and unique gene expression responses to the pathology, and that these changes recapitulate the human AD brain with increased age [27]. Hemizygous 5xFAD/homozygous *Trem2*^R47H^ (*Trem2*^R47H^; 5xFAD) mice enable transcriptomic analysis of concerted *Trem2*^R47H^ and 5xFAD induced patterns of transcriptomic dysregulation across the brain.

In this study, we utilized MERFISH (Multiplexed Error-Robust Fluorescence In Situ Hybridization) on wild-type (WT), 5xFAD, *Trem2*^R47H^, and *Trem2*^R47H^; 5xFAD mice to probe spatial gene expression in single cells [28, 29]. MERFISH utilizes multiplexed fluorescence in situ hybridization to identify individual RNA transcripts at subcellular resolution, enabling spatial transcriptomic analysis of cell transcriptomes in relation to their spatial environment.

Previous studies using MERFISH have analyzed spatial transcriptomics of neurodegeneration in aging [30], and in microglia activation [31], proving the efficacy of MERFISH in investigating transcriptomic dysregulation in the brain. However, studies analyzing spatial impacts of Alzheimer’s disease in mouse models have been limited either in cell type-specific impact analysis [32], spatial resolution [33], or the size and number of imaged regions [24]. In contrast, we present here a single-cell resolution, spatial transcriptomic atlas of 5xFAD and *Trem2*^R47H^ induced alterations across whole mouse coronal sections. Our findings reveal spatially localized cell type-specific plaque and *Trem2*^R47H^ induced transcriptome dysregulations in both glia and neurons in multiple cortical and subcortical brain regions.

## Materials and methods

### Animals

The *Trem2*^R47H^ mice used in this study were derived from the same *Trem2*^R47H NSS^ (Normal Splice Site) mouse colony with C57BL/6 J background as previously reported (Jax stock #034036 [22]). This same genetic background was used for all animals in this experiment. All animals were bred and raised by the Transgenic Mouse Facility at UCI under a regular light/dark (12 h/12 h) cycle with *ad libitum* access to food and water. All animal care and related experimental procedures were conducted following the highest ethical standards and were approved by the UC Irvine Institutional Animal Care and Use Committee.

### Preparation of mouse brain sections and MERFISH

Mice (wild-type, Trem2^R47H^, 5xFAD, and 5xFAD; Trem2^R47H^) were euthanized at 12 months of age via carbon dioxide inhalation followed by transcardiac perfusion with chilled phosphate-buffered solution (PBS, pH7.2). Brains were quickly collected with hemispheres bisected along the midline and separately embedded with another hemisphere from the designated genotype pair (WT with 5xFAD and Trem2^R47H^ with 5xFAD;Trem2^R47H^) in a square tissue mode with Tissue-Tek^®^ OCT mounting medium. Each pair of brains was flash frozen in dry ice-chilled isopentane and stored at −80 °C until cutting.

To prepare cryosections for MERFISH, two hemisphere OCT blocks containing 4 samples were combined and sectioned at −20 °C on a Leica CM1850 cryostat. A 10-µm-thick coronal slice containing both hippocampus and subiculum regions was collected onto a specially coated 4cm-diameter coverslip (Vizgen Item# 10500001), fixed in 4% paraformaldehyde in PBS in a 6 cm petri dish, and stored in 70% ethanol at 4 °C until MERFISH probe hybridization after a brief rinse with PBS.

MERFISH was performed according to Vizgen’s protocol. Briefly, merslides with mouse brain sections were rinsed with Vizgen Sample Preparation Buffer (SPB) after the removal of 70% ethanol, incubated with Vizgen’s Formamide Wash Buffer (FWB, 30 min at 37 °C), hybridized with a customized mouse gene panel containing specific binary-coded probes for selected 300 mouse genes (VZG171, Supplementary Table 1) in a parafilm-sealed plate ( ~ 40 h at 37 °C), and washed with FWB twice (30 min at 47 °C). After the aspiration of FWB, the brain sections were embedded with Vizgen gel mix, incubated in the clearing mix (5 mL with 50 μL protease K, overnight at 37 °C), stained with Vizgen DAPI-poly(T) reagent from the Vizgen 300-gene imaging kit (10 min at RT) after rinse with SPB, and washed with FWB (10 min at RT). Subsequently, the merslide was thoroughly rinsed with SPB before being carefully assembled into a gasket chamber.

Once assembled, each merslide was then uploaded into the MERSCOPE for imaging. The MERFISH imaging was done on the MERSCOPE with a Vizgen 300-gene imaging kit after the addition of the imaging buffer activator and RNase inhibitor (100 μL) as stated in the manual. The imaging process was controlled by the Vizgen MERSCOPE program (Software version 231.220726.1530b) with the default settings (both polyT and DAPI channels “on”, scan thickness: 10 μm). Once MERFISH imaging process was completed, the output files were transferred for in-depth analysis with MERFISH Visualizer and our customized bioinformatic pipeline.

Imaging occurred in 5 total batches: (1) one 5xFAD animal (female) and one WT animal (female), two brain slices (technical replicates) each; (2) one *Trem2*^R47H^; 5xFAD animal (female) and one *Trem2*^R47H^ animal (male), two brain slices each; (3–5) one slice from each genotype, no duplicated animals. All animals in batch 3 through 5 were male. The WT sample in batch 5 failed imaging QC and was not included. The failed sample exhibited low transcripts per field of view (FOV) counts (cutoff 10,000), the hippocampal and cortical regions were cut off (e.g. were outside the imaged region), and most of the cortex and hippocampus cells that remained exhibited low total gene counts (cutoff 25 per cell). These criteria were established after production of the first batch of samples, in conjunction with other experiments, based on computed requirements for cell type delineation. Samples from technical replicates were aggregated for subsequent analysis.

All slices were taken within a 0.7 mm range on the anterior-posterior axis, centered at −2.7 mm relative to bregma. Higher precision was not possible while retaining the ability to mount multiple slices on the same merslide, a requirement for accurate differential expression due to batch effects.

No statistical methods were utilized to compute sample sizes, but our sample sizes are comparable or larger than other similar experiments [24]. Researchers were not blinded to mouse genotype.

The gene panel was constructed based on the commercially available 300-gene set from Vizgen, and it contains markers for high resolution identification of individual cell types, with emphasis on learning and memory associated genes in cortical and hippocampal cell types, activated glial markers associated with disease associated microglia, and disease associated astrocytes.

### Verification of Aβ plaques by co-staining with thioflavin S and 6E10 antibody

Once the MERFISH imaging process was completed, validation merslides were washed in 100% formamide for 15 min to remove fluorescent readout probes, rinsed with 1xPBS, and stained with 6E10 mouse anti- A*β*_1–16_ monoclonal antibody (BioLegend #SIG-39320, 1:500 dilution, 2 h at RT) followed by PBS washes (5 min x3) and co-staining with 0.5% thioflavin S, and AlexFluor594-tagged goat anti-mouse IgG (ThermoFisher #A-11005, 1:600 dilution), and DAPI (10μg/ml) for 30 min at room temperature. Then the merslides were re-assembled into a gasket chamber and immediately imaged using the Merscope Verification mode with the default settings for protein staining (DAPI, anti-mouse, and anti-rabbit channels “on”, scan thickness: 10 μm).

### Processing of MERFISH datasets

Following automated transcript decoding and error correction via the MERSCOPE software pipeline, individual cells were segmented using the machine learning model *cellpose* [34], which was custom trained on DAPI stained slices collected previously, captured at the same resolution. Next, transcripts were assigned to individual cells. Cells exhibiting volume more than 1800 µm^3^, or less than 50 transcripts per cell were removed.

Data was then processed using our Scanpy [35] based custom pipeline, namely, library size normalization, log transformation, regression of sequencing depth per cell as a confounding variable, standard scaling, PCA transformation, batch integration using harmony (integrated by batch but not genotype) [36], and UMAP dimensionality reduction. Next, marker genes were computed (scanpy’s *sc.pl.rank_genes_groups function*) and matched to cell type reference atlases including the Allen institute cortex and hippocampus dataset [37], and the mousebrain.org single-cell reference atlas [38]. Cell type annotations were refined and corrected using spatial coordinates, particularly ensuring cortical-layer-specific neuron types were correctly organized. As part of the quality control process, cell types were subclustered, and cell subclusters exhibiting markers for other (typically glial) cell types were excluded from analysis as contaminated.

Region annotation of major spatial domains was performed using a semisupervised approach based on superposition and manual annotation of cell types on the appropriate Allen mouse brain coronal atlas slice [39], guided by the spatially localized cell types for fine region selection, particularly in hippocampus.

Subclustering was performed by subsetting to the desired cell type and running the same pipeline on the individual cell types. Subclusters containing less than 5% of the total cells were excluded from analysis. Markers for identified subtypes were identified using *sc.pl.rank_genes_groups*. Genotype bias was computed by first normalizing the number of cells in each subcluster by the total number of cells contributed from that genotype, and then normalizing by genotype, to obtain cell type proportions in each subcluster. The following thresholds were used to identify genotype bias: if a single genotype proportion for a given subtype exceeded 33% (25% being uniform distribution), the subtype was considered upregulated in that genotype. Additionally, if the combined proportion of two genotypes exceeded 60% (50% being uniform distribution), the subtype was considered upregulated in that pair of genotypes. This latter was restricted to identify only *Trem2*^R47H^, and 5xFAD specific upregulation (e.g. any subtypes co-upregulated in WT and *Trem2*^R47H^; 5xFAD were not considered for further analysis).

### Pseudobulk differential expression analysis

Cells were divided by cluster, genotype and batch. Genes present in fewer than 15% of cells were not analyzed for differential expression, due to limited accuracy of differential expression in low frequency genes [40]. Data for each cell type was aggregated by genotype and batch to construct pseudobulk replicates [41]. Samples with fewer than 50 cells of a given cell type were removed.

Due to unbalanced genotype proportions in individual batches, pairwise differential expression was performed separately for each genotype pair studied. Subsetting pseudobulk replicates to those associated with the compared genotypes, we utilized a linear mixed effects model (lme4 and multcomp packages [42, 43]), with batch as the random effect. Gene significance was identified using an absolute log fold change of 0.35, and an adjusted *p*-value of 0.05. The Benjamini-Hochberg method [44] was used for p-value correction. Fold changes were computed based on the inferred values by the LME model. Log fold changes were computed using a base of 2, unless otherwise stated.

### Continuous plaque proximity differential expression

Continuous plaque proximal differential expression was computed at the single-cell level for all cell types. Omitting the conversion to pseudobulk profiles, DESeq2 [45] was utilized to analyze differential expression contiguous to plaques, by including distance to plaque as a continuous covariate, and computing differential expression as a function of distance to plaque. Genotype was not used as factor in the model due to low sample numbers, and only 5xFAD and *Trem2*^R47H^; 5xFAD samples were utilized in this analysis. Only adjusted *p*-value (< 0.05) was used for identification of differentially expressed genes in this analysis.

### Differential expression between regions

To perform differential expression of glial cell types between different spatial regions in the same genotype, we utilized a pseudobulk approach. After subsetting to individual cell types and removing genes expressed in <15% of cells, we excluded sample-region combinations in which fewer than 50 cells are identified. We note that this completely excludes OPC analysis in the dentate gyrus due to lack of cells. At this point, we created pseudobulk samples for each sample-region combination (typically 10 pseudobulk samples for each slice, based on the 10 annotated regions). We then computed differential expression within the same genotype, comparing expression in each region to the average expression in all other regions, to identify spatially variable genes. We utilized a linear mixed effects model, with batch as the random effect, and gene significance was identified using an absolute log fold change of 0.35, and a *p*–value of 0.05.

### Cellpose segmentation of plaques

Initial visualization of DAPI staining in merslides appeared to show plaque staining in addition to cell nuclei. We confirmed this on MERFISH-imaged 5xFAD merslides by costaining with DAPI, thioflavin S, and 6E10 antibody against Aβ. To segment Aβ plaques in the DAPI image, we utilized the cellpose GUI to identify plaques based on DAPI brightness, plaque size (frequently larger than cells), and the presence of fibrils and irregular cellular shape. We note that this methodology may not detect the smallest volume plaques, so our spatial plaque analysis focuses on the impact of large plaques on neuronal and glial gene expression.

We utilized the cellpose GUI to manually label DAPI stained plaques in 125 individual ROIs (2000 pixels x 2000 pixels; 0.108 μm per pixel), taken from two of the 5xFAD and *Trem2*^R47H^; 5xFAD samples. Of these, 80% were used for training, and 20% for validation. A custom cellpose model was trained on the training data, and error results reported based on the testing set. False positive and False negatives were manually identified in each test ROI as follows. Contiguous regions exhibiting the brightness, size and morphology characteristics noted previously were annotated via the cellpose GUI. These were compared with predicted plaque locations via the trained cellpose model. Predicted cells showing significant overlap with annotated regions in the test set were considered as true positives, while missed regions were labeled as false negatives, and predicted cells showing no overlap with annotated regions were considered false positives.

We analyzed false positive and false negative rates, as well as F1 scores (defined as $\frac{2* {true\; positives}}{2* {true\; positives}+{false\; positives}+{false\; negatives}}$), a metric which balances sensitivity and specificity, assigning a single score in range [0,1] for model quality. In testing the model on the hold out annotated plaque data, we identified only 1 false positive and 0 false negatives across 25 FOVs, and 28 total annotated plaques, resulting in a false positive rate of 0.035, a false negative rate of 0, and an F1 score of 0.89. As additional measures of accuracy, we visually inspected the identified plaques in comparison with cells and demonstrate that detected plaques exhibit significantly lower transcript counts than are expected from cells.

Next, we tested our model on a hold-out 5xFAD sample prepared using the same methodology as for our analyzed slices, but stained using DAPI, thioflavin S (canonical Aβ plaque stain) and 6E10 (amyloid beta monoclonal antibody) for comparison. We annotated plaques using the cellpose GUI cytoplasm model, setting mean diameter to the same value (205 pixels) as was used in the cellpose plaque model, utilizing the 6E10 stain results for segmentation. Identified plaques intersecting the boundary of the ROI were not considered.

We again tested the algorithm on 23 ROIs (2000 pixels x 2000 pixels; 2 ROIs were removed from a total of 25 test ROIs due to low quality) from this combined stain slice. This resulted in 73 annotated plaques, of which 76% were detected, along with one false positive (false positive rate 0.017), and an F1 score of 0.86. The reduced sensitivity was possibly caused by alterations in DAPI contrast ratios, which affected the algorithms predictive capabilities. Overall, the protein-stained slices exhibited lower quality, one reason the protein/antibody stains were not included in the generation of analyzed slices.

In this analysis, no plaques were identified with thioflavin S or 6E10 that did not also overlap with significant DAPI stains of similar size. In general, DAPI plaques were identified as slightly larger than ThioS plaques (average 18.6% greater area, *p* = 0.018, Wilcoxon rank sum test), although this may be due to variability in the thresholds used to define plaque boundaries.

### Filtering of differentially expressed genes

Due to irregular cellular shapes, cellular processes distal to the soma, microglia phagocytosis, and possible segmentation errors, overall upregulation in expression within one cell type, may overlap into another. This pattern was particularly noticeable in glia, which frequently exhibited expression overlap in marker genes, as well as neuronal specific genes (such as *Slc17a7*). As an example, *Gfap* was ubiquitously expressed, and differentially expressed in 5xFAD samples compared with WT in many neuronal cell types. Yet its expression is limited almost exclusively to astrocytes in previous mouse brain cell atlases [37, 38].

To filter out these false positive differentially expressed genes, we heavily annotated all major glia subtypes for gene expression of all 300 genes in the panel, using both the mousebrain.org [38] and Allen Institute datasets [37] (Supplementary Table 2). Initial annotation utilized the Allen institute dataset, requiring a trimmed mean expression greater than zero. This was supplemented using the mousebrain.org dataset, which is not restricted to the hippocampus and contains activated microglia and astrocyte cell types. Differential expression of glial cells was then subset to genes known to be expressed in these cell types as annotated. Differential expression in neurons was filtered for expression of glial cell type markers, and disease associated microglia and astrocyte markers. While we recognize this process may introduce bias into the differential expression results, raw results exhibited significant cell type-induced biases complicating analysis of results. Raw differential expression results for all cell types are available in the supplemental tables.

Additionally, hippocampal differential expression is known to be correlated with location on the anterior-posterior axis, which varies across samples [46]. Subclustering identified spatially localized subgroups for each cell type, primarily in the ventral hippocampus. Marker genes for these clusters were identified, and differentially expressed genes overlapping these subsets were removed from the analysis. We also compared gene expression with differentially expressed genes varying on spatial gradients on the anterior-posterior axis in all brain regions and removed differentially expressed genes previously identified with such spatial gradients (e.g. we removed any differentially expressed genes present in the top 600 predictive genes for anterior-posterior axis location [46]).

### Computation of cell density within major regions

To compute the area of identified regions in individual slices, we utilized the python package alphashape [47], a method for automatically constructing concave bounding envelopes of point clouds. For each region, we subset to all cells contained in that region, and utilized alphashape with an alpha of 0.015, and computed the area of the resulting polygon. The same process was utilized on a cell type basis to compute neuronal cell density on a cell type level in each slice. For individual cell types, spatial outliers were removed prior to area inference via computation of the k-nearest neighbors, and analysis of the 5th nearest neighbor, using a standard outlier removal technique of eliminating cells outside the range (median-1.5*IQR, median+1.5*IQR, IQR=interquartile range), applied to distance to the fifth nearest neighbor. This ensures that areas are computed only in densely packed cellular regions.

For computing the density of glia in regions proximal and distal to plaques, all cells in each individual slice within 100 µm (proximal) and between 100 and 500 µm (distal) were used to compute the combined area of all regions distal and proximal to plaques. This was then used as the normalizing factor to obtain cell densities proximal and distal to plaques. When investigating density of individual cell types, we used the region identified using all cells within 50 µm of a single cell of the given subtype, as some cell types were insufficiently dense for area inference via alpha shape.

### Statistical methods

Excluding differential expression (described above), statistical tests are described in the text. We utilized two-sided tests unless otherwise stated. Differential expression results are presented as adjusted p-values unless otherwise stated.

## Results

### Impact of 5xFAD and *Trem2*^R47H^ mutations across major brain cell types

**Fig. 1:**
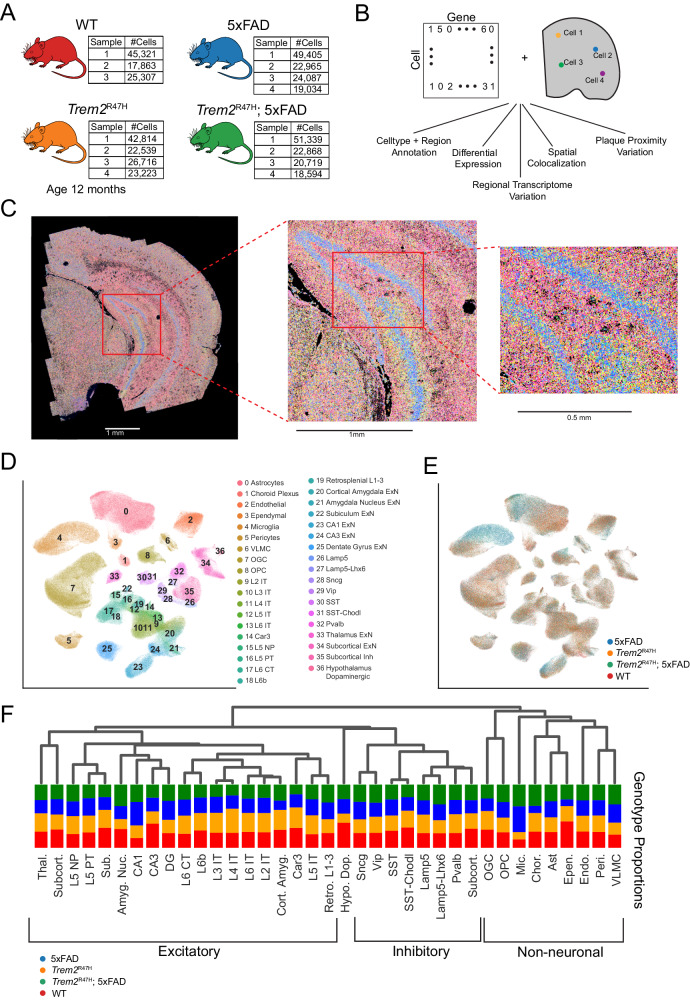
MERFISH spatial transcriptomics enables spatial variation analysis of the transcriptome at the cell type level. **A** Dataset overview consisting of 15 samples, from WT, Trem2^R47H^_,_ 5xFAD and *Trem2*^R47H^; 5xFAD mice. Cell counts in batch 1 are aggregated across two technical replicates, resulting in approximately double the cell counts of the other batches. **B** Integration of cell by gene matrix with RNA spatial location enables spatial analysis of transcriptomic variation on a regional and genotype basis. **C** 300 gene overlay on a single coronal section, at increasing resolutions. **D** UMAP displaying 37 annotated cell types after integration across all samples. **E** UMAP of cell genotypes. Note the distinct subpopulations specific to 5xFAD and Trem2^R47H^; 5xFAD genotypes, particularly in microglia and astrocyte cell populations. **F** Hierarchical organization of cell clusters, combined with raw cell type proportions per genotype.

In this study we investigate regional, plaque proximal, and genotype specific gene expression changes induced by the *Trem2*^R47H^ mutation. As this mutation is not sufficient to induce amyloid plaque pathology in mice, we utilize a hemizygous 5xFAD/homozygous *Trem2*^R47H^ mouse model which induces Aβ pathology in concert with the *Trem2*^R47H^ mutation, compared with matched 5xFAD (Aβ pathology only), *Trem2*^R47H^, and WT controls (Fig. 1A). By comparing these four genotypes, we uncover the transcriptomic alterations induced specifically by 5xFAD transgenes (independent of *Trem2*^R47H^ mutation), specifically by *Trem2*^R47H^ (independent of 5xFAD), and those induced by a combination of 5xFAD and *Trem2*^R47H^ mutations.

We performed spatial transcriptomic analysis using MERFISH on 19 coronal half sections from 15 total animals with WT, 5xFAD, *Trem2*^R47H^ and *Trem2*^R47H^; 5xFAD genotypes at 12 months of age, which age was chosen to match the later timepoint in our previous studies, which we have extensively characterized [22, 27]. Mice at this age exhibit an extensive plaque burden throughout the brain, combined with neuritic damage and glial responses.

After quality control, this dataset resulted in 432,794 cells. Using a 300 gene panel, we identified 37 major cell types, and transcriptomically and spatially mapped 5xFAD and *Trem2*^R47H^ transcriptomic alterations at the single-cell level. We also identified Aβ plaque locations in the same samples and assessed their relationship to spatial gene expression (Fig. 1B).

After spatial transcript decoding (Fig. 1C), cells were processed using our single-cell pipeline (Supplementary Fig. 1A, B), and clusters were identified based on reference to known cell type markers (Supplementary Fig. 1C), in conjunction with spatial location (Fig. 1D). Color coding genotype information on the UMAP shows strong 5xFAD induced cell type composition changes, reaching significance only in microglia (*p* = 0.0012, Wilcoxon rank sum test) (Fig. 1E). Hierarchical clustering identified initial splits between non-neuronal and neuronal cells, followed by excitatory vs. inhibitory, and spatial (subcortical, hippocampal, cortical) based splits in excitatory cell types (Fig. 1F).

**Fig. 2:**
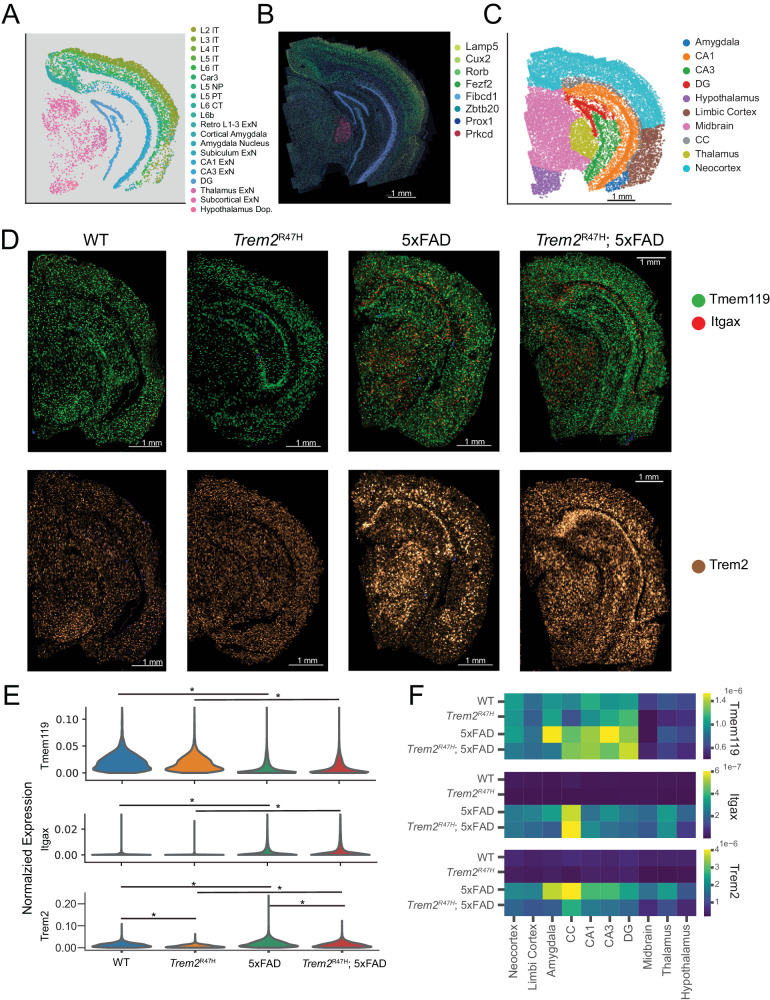
Spatial and transcriptomic analysis of coronal brain slices enables analysis of the spatial distribution of individual genes. **A** Spatial position of neuron subpopulations from single coronal sample. **B** Raw transcript overlay on PolyT cell body staining, of cell type markers for a subset of the neuron subpopulations in **A**. **C** Annotated spatial regions for a single coronal sample. Annotation performed based on transcriptomic cellular locations, combined with the Allen mouse brain reference atlas. **D** Raw transcript overlays of *Tmem119* (homeostatic microglia), *Itgax* (disease associated microglia), and *Trem2* on top of DAPI nuclei staining (blue). Point brightness indicates pixels where multiple individual transcripts were aggregated. **E** Violin plots of normalized expression of the genes indicated in **D**, divided by genotype and aggregated across samples. Asterisks indicate statistical significance (*p* < 0.05, linear mixed effects model). **F** Regional mean normalized expression in microglia aggregated within genotypes.

Visualization of neuronal cell types (Fig. 2A, Supplementary Fig. 2) show strong spatial localization, commensurate with previous region-based studies and atlases. Hippocampal excitatory cells define the primary structures of the hippocampal formation (DG, CA1, CA3), while cortical excitatory neurons divide into distinct layers across most of the cortex. We identified and visualized cell type-specific markers for these distinct neuron types (Fig. 2B) to verify spatial fidelity with raw decoded transcripts.

Next, we segmented major brain regions, subdividing the cortex into three subregions: the neocortex (somatosensory, visual, parietal, retrosplenial, and auditory cortices), the limbic cortex (perirhinal, ectorhinal, entorhinal, and piriform cortices), and the cortical amygdala, and identify major structures in hippocampal and subcortical regions. This resulted in 10 identified major brain regions (Fig. 2C).

We visualized raw transcript counts of *Tmem119* and *Itgax* to confirm microglia activation in the 5xFAD and *Trem2*^R47H^; 5xFAD mice. *Tmem119* is a homeostatic microglia marker, while *Itgax* is a marker for disease associated microglia (DAM), a distinctive microglia subset whose activation is associated with neuroinflammatory responses, including response to Aβ plaque pathology. As expected, 5xFAD and *Trem2*^R47H^; 5xFAD mice show *Itgax* expression upregulation, indicating increased microglial activation (Fig. 2D–E, *Itgax: p* < 0.02, *Tmem119: p* < 10^−10^, linear mixed effects model). Microglia transition to a fully activated state via a two-stage *Trem2* dependent pathway, highlighting the importance of this gene in AD progression [48]. We note that *Trem2* expression is significantly increased in the microglia of both 5xFAD and *Trem2*^R47H^; 5xFAD mice (Fig. 2E, 5xFAD: adjusted *p* = 2.6 × 10^−3^, fold change = 1.88, Trem2^R47H^; 5xFAD: adjusted *p* = 7.1 × 10^−6^, fold change = 1.89. Linear mixed effects model). We also note that *Itgax* and *Trem2* expression is consistently upregulated in 5xFAD and *Trem2*^R47H^; 5xFAD mice across most regions (Fig. 2F).

Finally, we analyzed neuronal density for each cell type by computing the total number of detected neurons divided by the estimated volume of the associated regions. No statistically significant trend was identified, however this may be due to our relatively small number of sample sections per genotype and the section variance across samples.

Overall, MERFISH spatial transcriptomics enables detection of high-level cell type clusters, visually identifiable and quantifiable transcriptomic differences in microglia and regional annotation and assignment of individual cells to specific coarse-grained spatial regions.

### Glial and neuronal transcriptomes are affected by nearby plaques

**Fig. 3:**
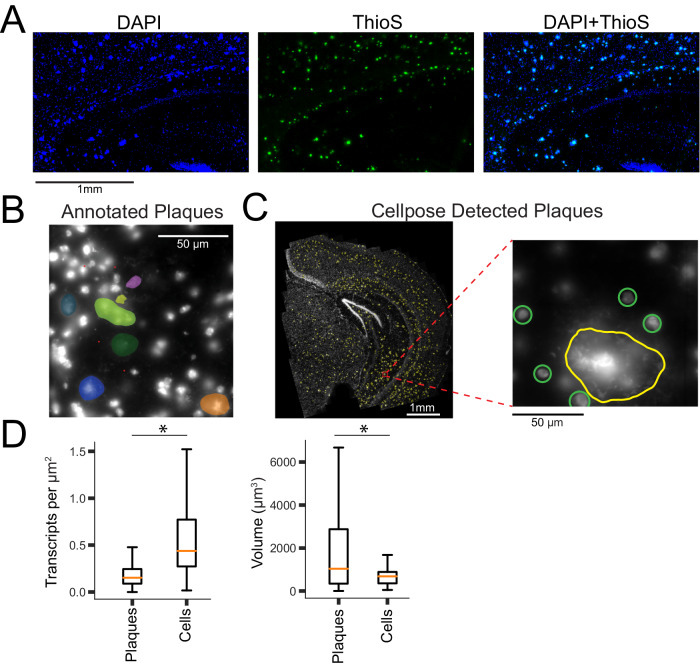
Machine learning enables accurate identification of plaque locations across brain slices. **A** DAPI (nuclei staining), ThioS (plaque staining) and overlay indicate that DAPI stains both nuclei and plaques. **B** Manual annotation of Aβ plaques (differentiated from cells by size, brightness, and morphology) is used as the basis for a machine learning model to detect plaque locations. **C** Detected plaques (yellow) in a single 5xFAD sample. Zooming in (right panel) we see that the machine learning model identifies the plaque, but not the cells surrounding it (manually circled, green). **D** Plaques exhibit significantly lower transcript density than cells (*p* < 0.0001, *t*-test), and significantly higher volume (*p* < 0.0001, *t*-test).

Spatial transcriptomics can reveal local effects of pathology, such as Aβ plaques, on the regulation of gene expression in nearby cells. By co-staining coronal brain slices with both DAPI and thioflavin S (a canonical stain for Aβ plaques), we observed that DAPI brightly labels Aβ plaques in addition to nuclei [49] (Fig. 3A). We therefore applied DAPI staining to MERFISH prepared coronal slices and a machine learning approach to automatically detect and segment plaques in each of the MERFISH samples.

DAPI stained plaques are visually distinguishable from nuclei by their large size, greater brightness, and fibrous morphology and lack of circular cell soma shape (Fig. 3B, C). These features enable manual annotation of plaques in individual fields of view. We trained a modified cellpose model [34] to detect large plaques (mean diameter 22.4 µm), but not cells (mean diameter 8.5 µm) (Fig. 3B, Supplementary Fig. 3A, B).

We analyzed each 5xFAD and *Trem2*^R47H^; 5xFAD section using this model (Supplementary Fig. 3C, D) and verified that (1) the model does not detect cells (Fig. 3B), (2) the predicted plaques are morphologically distinct from cells (Fig. 3C), and (3) the predicted plaques have significantly lower transcript density when compared to cells (Fig. 3D, *t*-test, *p* < 0.0001), as well as greater volume (*t*-test, *p* < .0001). Across all 5xFAD and *Trem2*^R47H^; 5xFAD samples, we identified a total of 5616 plaques (per sample: 5xFAD- 659.2 ± 160.1, *Trem2*^R47H^; 5xFAD- 464 ± 146.9, mean ± s.e.).

**Fig. 4:**
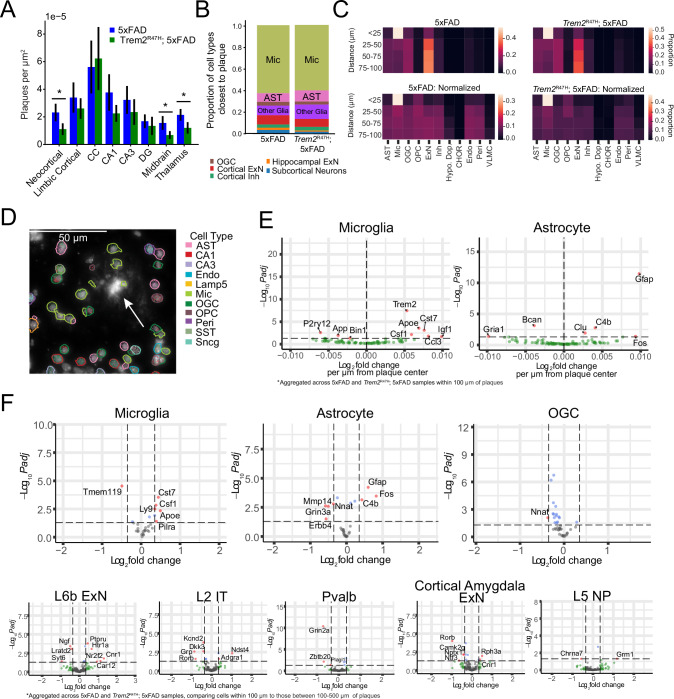
Aβ plaque proximity causes transcriptomic dysregulation in both glia and neuronal cell types. **A** Example plaque (arrow) with associated annotated cell types. Note the congregation of microglia around the plaque. **B** Proportion of cell types identified as closest to plaques. For each plaque, the closest cell was identified, and the proportion of resulting cell types was computed. **C** Cell type proportions within annuli at specific distances from plaque centers. The top row indicates raw cell proportions, while the bottom row shows cell type proportions after normalization by the total number of cells in that group. **D** Aβ plaque density by region. Statistical comparison (*p* < 0.01, linear mixed effects model) identifies three regions with significantly lower plaque density in *Trem2*^R47H^; 5xFAD animals. **E** Differential expression results for microglia and astrocytes using distance to plaque as the continuous dependent variable. Cells were selected such that all tested cells were within 100 µm of the center of a plaque. Cells aggregated across 5xFAD and Trem2^R47H^; 5xFAD samples. Genes filtered by expression in the associated cell type as identified in previous studies. No other cell types exhibited more than one differentially expressed gene in this test. Results indicate expected expression changes per µm distance increase from closest plaque center. **F** Differential expression results testing cells within 100 µm of the center of a plaque against those 100–500 µm from the center of a plaque. The cell types with the largest number of DE genes among glia and neurons are visualized here. Genes filtered by expression in the associated cell type as identified in previous studies. Red points indicate genes exceeding both adjusted p-value and log fold change thresholds, green points only exceed log fold change thresholds, blue points only exceed adjusted *p* threshold, and gray points exceed no threshold.

Across brain regions, we found the closest cell to each identified plaque was most frequently microglial (62.6% of plaques in 5xFAD and 60.0% in *Trem2*^R47H^; 5xFAD) (Fig. 4A). Additionally, microglia density in the region within 100 µm of a plaque (proximal) was significantly higher than in the 100–500 µm region(distal) (5xFAD, proximal density = 17.6 ± .935 × 10^−5^, distal density = 7.81 ± 0.665 × 10^−5^, *p* = 7.55 × 10^−4^; *Trem2*^R47H^; 5xFAD, proximal density = 17.6 ± 0.790 × 10^−5^, distal density = 6.87 ± 2.15 × 10^−5^, 1.31 × 10^−3^, mean ± s.e, plaques/µm^2^), while no genotype difference was detected for density either proximal or distal to plaques (*p* > 0.19). Astrocytes were the second most common cell type identified near plaques (7.9% of plaques in 5xFAD and 10.1% in *Trem2*^R47H^; 5xFAD) (Fig. 4A), however, overall astrocyte density showed no differences in density between proximal or distal areas in either genotype (*p* > 0.16). Thus, the typical microenvironment around plaques includes microglia, with astrocytes and other cell types at greater distances from the plaque (Fig. 4B) [24, 50]. We then analyzed cell type proportions in annuli around individual plaques measured at 25 µm intervals. The smallest 25 µm circle around each plaque center was populated almost exclusively by microglia, with other cell types becoming more prevalent with increasing distance to plaque (Fig. 4C).

We assessed whether plaques appear proximal to neurons. The distance from a plaque to the closest neuron was significantly larger than the distance from a neuron to its closest neuronal neighbor (5xFAD: minimal plaque to neuron distance 56.4 ± 10.7 µm, minimal neuron to neuron distance 21.6 ± 1.24 µm, *p* = 0.012; *Trem2*^R47H^; 5xFAD: minimal plaque to neuron distance 48.7 ± 2.53 µm, minimal neuron to neuron distance 21.0 ± 0.733 µm, *p* = 1.47 × 10^−4^, mean ± s.e., plaques in corpus callosum excluded from analysis due to lack of nearby neurons, *t*-test). We examined the typical distance of each neuronal cell type to the nearest plaque. This analysis showed that subiculum, layer 5, and layer 6 excitatory neurons have the lowest median distance to plaques among identified cell types (Supplementary Fig. 3E). However, none of the top 5 neuron types (ranked by median distance to plaque, excluding subiculum excitatory and SST-Chodl cells due to low cell numbers), exhibited significant density variation between plaque proximal (<100 µm) and distal (100–500 µm) regions. This implies that neuronal plaque proximity is driven primarily by plaque density in the associated regions. Additionally, plaques on average form in regions nearly twice as far from the nearest neuron as the typical distance between neurons, but the average neuronal density does not appear to be decreased in plaque proximal vs. plaque distal regions, implying a variation in plaque to neuron distance at the microscale (<100 µm), but not at larger scales (<500 µm).

The highest plaque density occurred in the corpus callosum (CC) (5xFAD average 5.61 × 10^−5^, *Trem2*^R47H^; 5xFAD average 6.23 × 10^−5^ plaques/µm^2^) and hippocampal areas (5xFAD average 2.89 × 10^−5^, *Trem2*^R47H^; 5xFAD average 2.04 × 10^−5^ plaques/µm^2^, averaged across CA1, CA3, and DG), followed by cortex (5xFAD average 3.92 × 10^−5^, *Trem2*^R47H^; 5xFAD average 2.08 × 10^−5^ plaques/µm2, averaged across neocortex, limbic cortex, and cortical amygdala), with the lowest densities in the subcortical regions (5xFAD average 2.71 × 10^−5^, *Trem2*^R47H^; 5xFAD average 0.323 × 10^−5^ plaques/µm^2^, averaged across midbrain, thalamus and hypothalamus) (Fig. 4D). Mice with the 5xFAD genotype had higher plaque density compared with *Trem2*^R47H^; 5xFAD mice in the midbrain, thalamus, and neocortex (*p* < 0.05, linear mixed effects model), but not the CC. This distribution is consistent with the pattern of median minimum distance to plaques (Supplementary Fig. 3E), with roughly all cell types showing larger distance to plaques in *Trem2*^R47H^; 5xFAD samples. High plaque density regions such as the subiculum and lower cortical layers contained neurons with the lowest median distance to the nearest plaque. *Trem2*^R47H^; 5xFAD animals exhibited larger plaque sizes than 5xFAD animals (1108 µm^3^ vs. 984 µm^3^, *p* = 0.025, Wilcoxon rank sum test), though this appears to be gender and pathology dependent, as male animals showed the reverse effect (741.96 µm^3^ vs. 799.60 µm^3^, *p* = 0.0046) as well as lower pathology levels (Supplementary Fig. 3C, D).

We next tested whether cells within 100 µm of the nearest Aβ plaque have altered patterns of gene expression (Fig. 4E). Due to the relatively low cell abundance proximal to plaques, we aggregated 5xFAD and *Trem2*^R47H^; 5xFAD samples, and separated individual cells by cluster. We analyzed plaque proximity based differential expression with two techniques. First, we identified cells within 100 µm of a plaque center. Using DESeq2 and treating cells as independent samples, we identified genes whose expression correlated with proximity to the nearest plaque (Fig. 4E, Supplementary Table 3). Continuous effects were identified primarily in microglia and astrocytes, with microglia showing an upregulation of typical DAM associated genes (e.g. *Csf1, Apoe, Cst7*), and a downregulation of *P2ry12*, a homeostatic microglia associated gene [51]. Similarly, *C4b*, *Clu*, and *Gfap*, markers of a previously known disease associated astrocyte (DAA) phenotype were also upregulated near plaques [52].

To validate these findings and to account for variability across biological replicates, we additionally performed a pseudobulk analysis of differential expression between plaque-proximal (within 100 µm of the closest plaque) and plaque-distal (100–500 µm to closest plaque). We applied a linear mixed effects model to pseudobulk expression for each cell type in each sample, accounting for batch as a random effect (Fig. 4F, Supplementary Table 4). Additionally, we filtered genes based on their known expression in each cell type from previous single-cell atlases [37, 38], to avoid spurious identification of differentially expressed genes due to technical (errors in segmentation) or biological (phagocytosis, overlapping cellular processes) effects [53].

Pseudobulk analysis was generally consistent with the DESeq2 results and identified both glial and neuronal changes (Fig. 4F). Microglia and astrocytes exhibited typical disease associated profiles in cells located proximal (<100 µm) to plaque centers. However, *Nnat* expression in astrocytes and oligodendrocytes, and *Mmp14* expression in astrocytes decreased near plaques. This result contrasts with previous findings in humans and other mouse models showing upregulation of *Mmp14* in reactive astrocytes in AD [54].

The pseudobulk analysis also revealed notable changes in gene expression affecting neurons proximal to plaques (Fig. 4F, bottom row). L6b neurons showed lower *Ngf* expression near plaques, a gene therapy target in AD [55]. *Nr2f2*, upregulated near plaques, is known to be dysregulated by AD associated single nucleotide polymorphisms in the *APOE* enhancer [56]. Cnr1 and Htr1a, also upregulated near plaques, are linked to regulation of the serotonergic system, which is known to affect memory in the context of AD [57]. L2 intratelencephalic (IT) neurons near plaques showed downregulation of *Dkk3* (a WNT signaling modulator whose presence reduces Aβ pathology in mouse models [58]) and of the potassium ion channel subunit *Kcnd2* [59] near plaques. L5 NP cells show *Grm1* upregulation and *Chrna7* downregulation near plaques. Parvalbumin-expressing inhibitory cells shows *Grin2a*, *Zbtb20*, and *Plagl1* downregulation near plaques. Excitatory neurons in the cortical amygdala exhibited downregulation of *Ntf3* (associated with nervous system maintenance [60]), *Nptx1* (associated with synapse remodeling, but typically upregulated in previous studies of cortical neurons near plaques [61]), and *Camk2g* (implicated in synaptic plasticity [62]). Because there were few plaques in subcortical regions, we did not test plaque-associated differential expression for neuronal cell types in this region.

### Microglia and astrocytes exhibit distinct cell type-specific spatial patterns of activation associated with 5xFAD mutation

We next directly analyzed spatial and transcriptomic variation of glia between genotypes. We made four pairwise comparisons (5xFAD vs. WT, *Trem2*^R47H^; 5xFAD vs. *Trem2*^R47H^, *Trem2*^R47H^ vs. WT, *Trem2*^R47H^; 5xFAD vs. 5xFAD), to identify 5xFAD and *Trem2*^R47H^ dependent variations, which we then compared with differential expression between *Trem2*^R47H^; 5xFAD and WT (Supplementary Table 5).

**Fig. 5:**
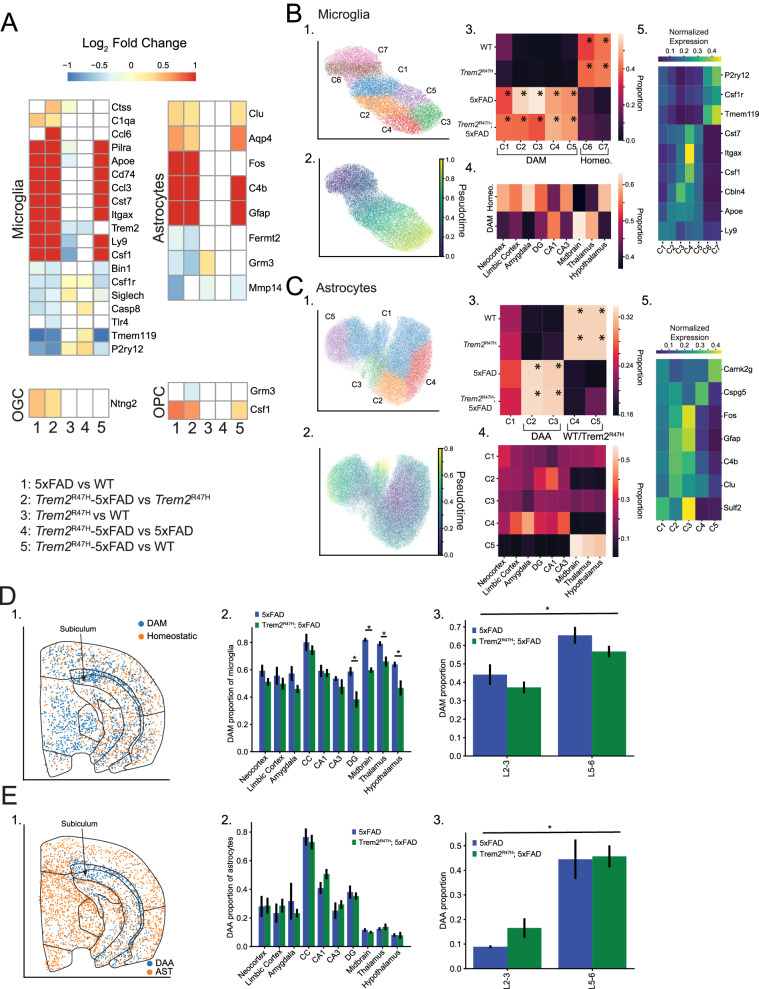
Microglia and astrocytes exhibit 5xFAD induced transcriptome alterations. **A** Pairwise differential expression between genotypes among glia populations. Five pairwise comparisons are indicated (5xFAD vs. WT, *Trem2*^R47H^; 5xFAD vs. *Trem2*^R47H^, *Trem2*^R47H^ vs. WT, *Trem2*^R47H^; 5xFAD vs. 5xFAD, *Trem2*^R47H^; 5xFAD vs. WT). Heatmaps display log fold change for each comparison, with genes not exceeding significance set to 0. Heatmaps are thresholded to the range (−1, 1). Genes not exhibiting significant expression in the associated cell type according to the Allen or mousebrain references were removed. Cell types exhibiting no differentially expressed genes not shown. **B** (1) Subclustering results for microglia. Clusters with transcriptomes influenced by spatial colocalization with other cell types removed. (2) Diffusion pseudotime results, indicating a non-bifurcating differentiation trajectory. (3) Genotype proportions for each subcluster. Clusters C1-4 are found primarily in 5xFAD and *Trem2*^R47H^; 5xFAD mice, with C6-7 localized to WT and *Trem2*^R47H^ animals. Asterisked clusters pass threshold for overabundance. Results indicate the pseudotime trajectory (1) describes a genotype specific transition. (4) Proportions of DAM and homeostatic microglia within annotated regions. Significant variations in distribution include decreased DAM proportions in cortex, DG, CA3 and hypothalamus. (5) Upregulated genes in each subcluster divide into homeostatic and DAM associated genes. **C** Astrocyte subclustering analysis. (1) Subclustering results, unbiased (by genotype proportion) clusters combined and relabeled as C1. (2) Pseudotime trajectories indicate no clear differentiation pattern. (3) C2-3 exhibit 5xFAD and *Trem2*^R47H^; 5xFAD upregulation, with C4-5 upregulated in WT and *Trem2*^R47H^ mice. (4) Significant spatial variation indicates C5 and C4 are differentiated by spatial location (subcortical vs. cortex/hippocampus), while C2 also appears upregulated in Cortex and Hippocampus, and C3 is distributed evenly across regions. (5) C2-3 exhibit upregulation of *C4b* and *Gfap*, part of the disease associated astrocyte (DAA) phenotype, while the spatially variable C4 and C5 differentiate by *Cspg5* and *Camk2g* expression. **D** (1) spatial distribution of DAM and homeostatic microglia overlaid on a 5xFAD sample. (2) DAM proportion of total microglia in each region. Error bars indicate standard errors. Asterisks indicate regional differences between 5xFAD and *Trem2*^R47H^; 5xFAD mice (*p* < 0.05, linear mixed effects model). Highest concentrations of DAM in CC, midbrain, and thalamus. (3) DAM proportions of microglia divided by cortical layer. No statistically significant change detected between 5xFAD and *Trem2*^R47H^; 5xFAD mice, but statistically significant increases in DAM proportion in lower cortical layers. **E** (1) spatial distribution of DAA and homeostatic astrocytes overlaid on a 5xFAD sample. (2) DAA proportion of total microglia in each region. Error bars indicate standard errors. No statistically significant variations identified between genotypes. Highest concentrations of DAA in CC and surrounding regions. (3) DAA proportions of microglia divided by cortical layer. No statistically significant change detected between 5xFAD and *Trem2*^R47H^; 5xFAD mice, but statistically significant increases in DAA proportion in lower cortical layers.

We identified 19 differentially expressed genes in microglia and 8 in astrocytes across all four pairwise comparisons. By contrast, we found 1–2 differentially expressed genes in oligodendrocyte (OGC) and oligodendrocyte precursor cells (OPC) cell populations (Fig. 5A), and none in the other non-neuronal cell types. Microglia and astrocytes primarily exhibited 5xFAD dependent changes (similar differential expression results for both 5xFAD vs. WT, and *Trem2*^R47H^; 5xFAD vs. *Trem2*^R47H^), replicating the DAM/DAA gene upregulation and homeostatic gene downregulation identified in the plaque proximity analysis. These were widely replicated in differential comparison of *Trem2*^R47H^; 5xFAD and WT. Interestingly, neither *Itgax* nor *Cd74* were identified as differentially expressed in plaque proximity analysis of microglia, whereas they were upregulated 9.58 and 15.7-fold in 5xFAD compared with WT animals, and 19.7 and 26.0-fold in *Trem2*^R47H^; 5xFAD compared with *Trem2*^R47H^ animals. The *Trem2* gene itself showed a small reduction in expression dependent on the *Trem2*^R47H^ mutation, which we hypothesize may be due to reduced binding efficiency of gene probes overlapping the mutated region, as the effect was not seen in our previous study [22]. Differential expression also shows a small but significant Trem2^R47H^ specific upregulation in homeostatic microglia genes, including *Tmem119* (fold change = 1.13, adjusted *p* = 0.019, *Trem2*^R47H^; 5xFAD vs. 5xFAD), and *P2ry12* (fold change = 1.27, adjusted *p* = 4.60 × 10^−4^, *Trem2*^R47H^; 5xFAD vs. 5xFAD). The consistent variation in both *Trem2*^R47H^ vs. WT and *Trem2*^R47H^; 5xFAD vs. 5xFAD comparisons indicates this may be a plaque independent effect and corroborates the overall lower plaque burden in *Trem2*^R47H^; 5xFAD samples.

To explore the effects of AD risk genes in specific glial subtypes, we subclustered the microglia and astrocyte subpopulations. We identified several small clusters of microglia that appear to express neuronal or other glial markers, and we confirmed that these cells are located near cells expressing these markers. We removed these cells from this portion of the analysis. After removal, subclustering identifies 7 microglia clusters (Fig. 5B1). Pseudotime analysis identified a single linear trajectory across all microglial cell types (Fig. 5B2). We next examined the genotype proportions of these clusters. After normalizing by the number of cells per sample, we averaged across samples of the same genotype, and computed cluster proportions. This identifies a clear 5xFAD dependent bias, with two clusters (labeled homeostatic) exhibiting > 80% proportion coming from non 5xFAD (i.e. WT and *Trem2*^R47H^) samples. The remaining five clusters corresponded to disease associated microglia (DAM) enriched in 5xFAD and *Trem2*^R47H^; 5xFAD mice (Fig. 5B3).

We aggregated homeostatic and DAM subgroupings and identified regional spatial biases (Fig. 5B4). DAMs were enriched in hippocampal area CA1. They were also enriched in thalamus, and midbrain, despite the relative lack of plaque density in these regions compared to the CA1 and CC (Fig. 4D). Finally, we identified markers for the individual microglia subpopulations, and plot normalized expression (Fig. 5B5).

We focused on the analysis of the genes differentially associated with late-stage DAMs as several genes exclusive to late-stage DAM (DAM II) were included (*Itgax, Cst7, Csf1, Ccl6*), as well as genes present across both stages (*Apoe*). Except for *Ccl6*, all of these genes are differentially expressed in 5xFAD and *Trem2*^R47H^; 5xFAD, with primary expression of DAM2 genes in C3-5 (later pseudotime). *Apoe* is evenly distributed across C2-5, reflecting its overexpression across the DAM developmental timeline (Fig. 5B5) [63]. While we do not have explicit genes that encode DAM I compared with DAM II in the gene panel, clusters C1-2 likely contain DAM I microglia, based on the pseudodevelopmental timeline.

Subclustering the astrocyte subpopulations, we aggregated clusters not exhibiting genotype specific bias (see methods for thresholds) into a single cluster (C1), retaining the genotype biased clusters (Fig. 5C1). Pseudotime trajectory analysis (Fig. 5C2) did not yield a distinctive pattern, however after analysis of genotype bias (identifying C1 as unbiased, C2/C3 as DAA, and C4/C5 as upregulated in WT/*Trem2*^R47H^ samples, Fig. 5C3), we note that C5 and C4 exhibited distinct spatial distributions, with C4 appearing exclusively in cortex and hippocampus, and C5 appearing in subcortical regions (Fig. 5C4). The DAA exhibited a similar regional specificity, with C2 primarily restricted to cortex and hippocampus. Cluster markers are identified and plotted (Fig. 5C5).

We next examined the spatial distribution of DAM and DAA cells by region. Disease associated microglia were enriched in the CC, subiculum and subcortical regions (Fig. 5D1). Computing the proportion of microglia identified as DAM by region (Fig. 5D2) showed similar proportions of DAMs between 5xFAD and *Trem2*^R47H^; 5xFAD samples by region, except in the DG, thalamus, midbrain, and hypothalamus. This corresponds with the plaque density bias in 5xFAD samples (Fig. 4D). In the cortex, we saw a significant increase in DAMs in the lower cortical layers (L5/L6) compared with the upper cortical layers (L2/L3) (*p* < 0.0001, linear mixed effects model, Fig. 5D3).

Disease associated astrocytes were concentrated in the CC and surrounding areas (Fig. 5E1-2). Virtually no disease associated astrocytes were present in upper cortical layers, but this population was significantly upregulated in deeper cortical layers (Fig. 5E3). We did not find significant genotype specific effects in other glial cells.

To further analyze the spatial variation of gene expression, we performed direct pseudobulk differential expression analysis of microglia, astrocytes, oligodendrocytes and oligo-precursors across the 10 identified major brain regions (Supplementary Fig. 4, Supplementary Table 6). We compared each region with the average across the remaining 9 regions. We also computed regional cell density and cell proportion for each of these cell types.

Analysis of microglia (Supplementary Fig. 4A) showed that ~60% of spatially variable genes were also differentially expressed across genotypes. For example, the canonical late-stage DAM markers *Cst7* and *Itgax* were significantly upregulated in the corpus callosum. A small number of other genes (*Ctss*, *C1qa, Zbtb20, Ly9, Tmem119*) had spatially variable patterns of expression that were consistent across WT and *Trem2*^R47H^ mice and dysregulated in 5xFAD and *Trem2*^R47H^; 5xFAD mice. Microglia cell populations also showed drastic increases in both cell proportion and density across all brain regions.

Astrocytes (Supplementary Fig. 4B) exhibited large numbers of spatially variable genes with consistent patterns of expression across all genotypes (e.g. *Erbb4, Nnat, Grin3a, Mmp14, Id4, Pax6*, etc). We also found spatial variation in several disease associated genes (*Aqp4, Gfap*). These spatial variations were primarily observed between cortical and subcortical (thalamus, midbrain, hypothalamus) regions. However, astrocytes exhibited little genotype specific cell proportion or density variations between regions.

Oligodendrocytes exhibited two separate gene groupings (Supplementary Fig. 4C). One set (*Snca, Dlg4, Nnat, Robo1, S100b, Ptgds*) showed spatially variable expression across multiple regions, particularly between cortical/hippocampal and subcortical regions. The other set of genes (*Adam10, Psen1, Olig1*, etc) is primarily upregulated in CC and downregulated in amygdala, with very little variation in other regions. This pattern is not 5xFAD or *Trem2*^R47H^ dependent and was observed even in WT oligodendrocytes cells. This second pattern is not replicated in oligodendrocyte precursor cells, though a significant cortex vs. subcortical divide is present in OPCs (Supplementary Fig. 4D). Neither cell type exhibits significant genotype dependent cell proportion changes within regions.

Overall, our data show that spatial variation in microglia and astrocyte gene expression is more affected by 5xFAD than by *Trem2*^R47H^. Both disease-associated microglia and astrocytes exhibit specific spatial distributions. DAMs were distributed across the coronal section, but concentrated in the CC and subcortical regions, and DAA were biased almost exclusively to the CC and surrounding regions. Regional transcriptional variations were primarily impacted in 5xFAD for microglia and astrocytes, and both 5xFAD and *Trem2*^R47H^ mutations were independent of regional variations in oligodendrocytes and oligodendrocyte precursors.

### Neurons exhibit complex transcriptomic impacts of 5xFAD and Trem2^R47H^ mutations

**Fig. 6:**
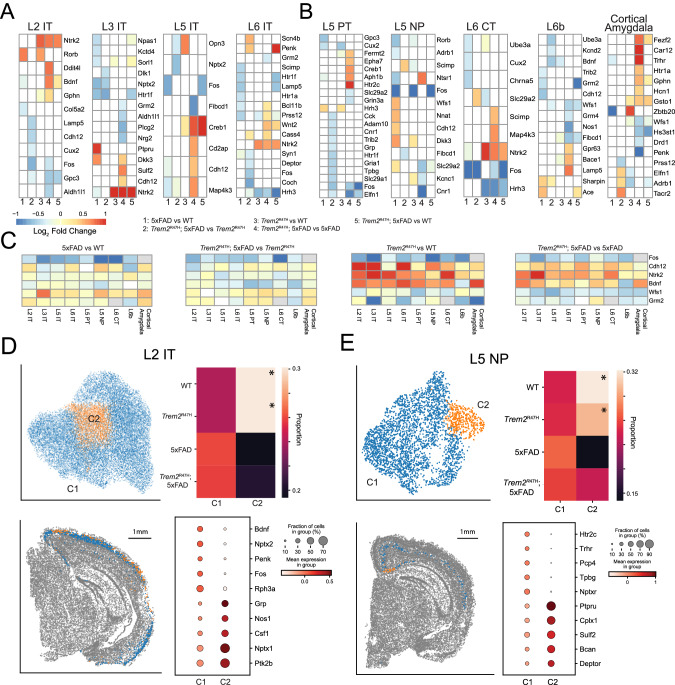
Cortical neurons exhibit consistent *Trem2* associated transcriptomic variations and spatially localized genotype biased subclusters. **A** Pseudobulk, linear mixed effects model differential expression results for cortical IT neurons. Heatmaps indicate log fold changes. Fold changes for genes not exhibiting significance set to 0 (white). **B** Differential expression results for other cortical excitatory cell types. **C** Log fold expression changes for each comparison for genes identified as consistently differentially expressed across multiple cell types. **D** Subclustering of L2 IT neurons (UMAP, top left) identifies a single subcluster overrepresented in WT and *Trem2*^R47H^ samples (top right, asterisked). This cluster is homogeneously distributed along layer 2 with bias for the neocortex (bottom left), and exhibits overrepresentation of *Grp*, *Nos1, Nptx1*, and *Ptk2b*. **E** Subclustering of L5 NP neurons (UMAP, top left) identifies a single subcluster overrepresented in WT and *Trem2*^R47H^ samples (top right, asterisked). This cluster is spatially localized to the retrosplenial cortex near the subiculum, an area of high plaque density. This cluster exhibits overrepresentation of *Ptpru, Cplx1, Sulf2*, and *Deptor*.

We performed differential expression analysis for each of the four comparisons (5xFAD vs. WT, *Trem2*^R47H^; 5xFAD vs. *Trem2*^R47H^, *Trem2*^R47H^ vs. WT, *Trem2*^R47H^; 5xFAD vs. 5xFAD), and compared with *Trem2*^R47H^; 5xFAD vs. WT differentially expressed genes, followed by subclustering analysis for each of the neuronal cell types (Supplementary Table 5). Analysis of cortical neurons identifies differentially expressed genes for all these comparisons in each cell type (Fig. 6A–C), as well as genotype biased subclusters for most neuron cell types (Fig. 6D, E, Supplementary Fig. 5). We first considered genes consistently identified as differentially expressed across multiple cortical neuronal cell types.

*Cdh12*, associated with calcium ion binding [64], was differentially expressed in 5 out of 9 cell types. Both L3 IT and L5 IT neurons exhibit upregulation of *Cdh12* in *Trem2*^R47H^; 5xFAD over 5xFAD genotypes. *Ntrk2*, which encodes TrkB, a high affinity receptor for BDNF [65], is upregulated in *Trem2*^R47H^; 5xFAD vs. 5xFAD and *Trem2*^R47H^ vs. WT comparisons in L2 IT, L3 IT, L6 IT and L6 CT excitatory cell types. On the other hand, *Bdnf* itself, expected to decrease in the 5xFAD context, was identified as significantly decreased only in L2 IT and L6b neurons. *Fos*, a molecular marker of neuron activity [66], was consistently identified as differentially expressed across 6 of the 9 cell types, for at least one comparison. In each case this gene was downregulated, implying downregulation of *Fos* induced by both 5xFAD and *Trem2*^R47H^ mutations.

All other genes were differentially expressed in at most 3 cortical excitatory cell types. Of these, the most interesting are *Wfs1*, recently implicated in Tau clearance in AD [67], which is downregulated in *Trem2*^R47H^; 5xFAD compared with 5xFAD, and *Grm2*, a glutamate receptor downregulated in *Trem2*^R47H^; 5xFAD compared with 5xFAD in L3 IT and L6 IT neurons.

We next examined log fold changes for the previously mentioned genes without applying statistical thresholds, to identify possible patterns across cell types obfuscated by our choice of threshold (Fig. 6C). *Cdh12* showed no consistent patterns in the 5xFAD comparisons but was consistently upregulated by the *Trem2*^R47H^ mutation (*Trem2*^R47H^ vs. WT: 0.591 ± 0.305, *p* = 3.79 × 10^−3^; *Trem2*^R47H^; 5xFAD vs. 5xFAD: 0.305 ± 0.197, 8.49 × 10^−4^; mean ± sd, computed average across cell types, *t*-test). *Ntrk2* exhibited a similar pattern (*Trem2*^R47H^ vs. WT: 0.706 +/− 0.366; *Trem2*^R47H^; 5xFAD vs. 5xFAD: 0.554 ± 0.330) and also showed a significant downregulation in the *Trem2*^R47H^; 5xFAD vs. *Trem2*^R47H^ comparison (−0.207 ± 0.133, *p* = 8.25 × 10^−4^). *Bdnf* did show a small decrease induced by 5xFAD (5xFAD vs. WT: −0.179 ± 0.132, *p* = 2.04 × 10^−3^; *Trem2*^R47H^; 5xFAD vs. *Trem2*^R47H^: −0.207 ± 0.133, *p* = 4.56 × 10^−4^), and interestingly, a consistent upregulation induced by *Trem2*^R47H^ (*Trem2*^R47H^ vs. WT: 0.581 ± 0.360, *p* = 6.50 × 10^−4^; *Trem2*^R47H^; 5xFAD vs. 5xFAD: 0.273 ± 0.374, *p* = 4.65 × 10^−2^), except in L5 PT and L6b neurons. Both *Wfs1* (*Trem2*^R47H^ vs. WT: −0.199 ± 0.209, *p* = 1.47 × 10^−2^; *Trem2*^R47H^; 5xFAD vs. 5xFAD: −0.448 ± 0.140, *p* = 3.18 × 10^−6^) and *Grm2* (*Trem2*^R47H^ vs. WT: −0.580 ± 0.481, *p* = 6.76 × 10^−3^; *Trem2*^R47H^; 5xFAD vs. 5xFAD: −0.234 ± 0.194, p = 6.91 × 10^−3^) were consistently downregulated by the *Trem2*^R47H^ mutation. *Fos* exhibits negative log fold changes in every cell type and comparison (5xFAD vs. WT: −0.778 ± 0.282, 3.47 × 10^−5^; *Trem2*^R47H^; 5xFAD vs. *Trem2*^R47H^: −0.522 ± 0.227, *p* = 7.96 × 10^−5^; *Trem2*^R47H^ vs. WT: −0.850 ± 0.370, *p* = 1.25 × 10^−4^; *Trem2*^R47H^; 5xFAD vs. 5xFAD: −0.552 ± 0.228, *p* = 8.57 × 10^−5^), indicating highly consistent activity downregulation induced by both 5xFAD and *Trem2*^R47H^ mutations.

Subclustering cortical neurons identified genotype-specific subpopulations in 7 cortical excitatory cell types (Fig. 6C–E, Supplementary Fig. 5). In all but one cell type (L3 IT), this represents a WT (or WT/*Trem2*^R47H^) enriched, and thus 5xFAD/*Trem2*^R47H^; 5xFAD reduced subpopulation. For most IT cell populations, these genotype specific subtypes did not exhibit significant spatial localization. However, 5xFAD/*Trem2*^R47H^; 5xFAD reduced subtypes in L2 IT are spatially localized to the retrosplenial and visual cortices, and 5xFAD/*Trem2*^R47H^; 5xFAD reduced subtypes in L5 NP are spatially localized in the retrosplenial cortex near the subiculum, a plaque dense environment (Fig. 6E, Supplementary Fig. 5). This latter subtype upregulated *Sulf2* (antibody staining has shown this is reduced in AD [68]) and *Cplx1* (regulates synaptic transmission by preventing neurotransmitter release prior to action potential [69]) as top differentially expressed genes.

**Fig. 7:**
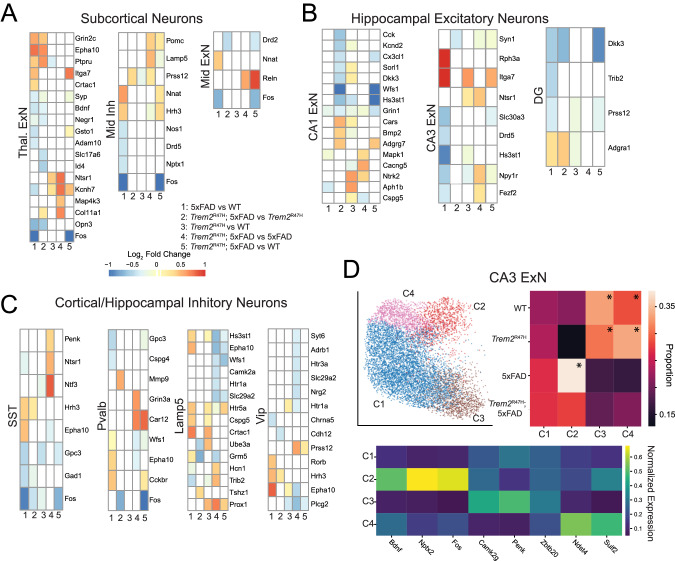
CA3 excitatory neurons exhibit significant 5xFAD induced transcriptional differences. Differential expression analysis of subcortical neurons **A**, Hippocampal excitatory neurons **B**, and Inhibitory interneurons **C**. Due to slice variation on the anterior to posterior axis, there are spatial biases in some cell types. Differential gene expression associated with possible spatial biases (as a confounding factor) are removed. Excitatory thalamic and CA1 excitatory neurons exhibit significant consistent variation on comparisons 1 and 2 (5xFAD vs. WT and Trem2^R47H^; 5xFAD vs. Trem2^R47H^, **A, B**). **D** Subclustering of CA3 excitatory neurons identifies a 5xFAD upregulated population (C2, asterisked) as well as two WT and Trem2^R47H^ upregulated populations (two additional subclusters were removed due to sample location bias).

Among subcortical neurons, thalamic excitatory neurons exhibited the largest number of differentially expressed genes (Fig. 7A). Thalamic excitatory neurons exhibit 5xFAD induced upregulation of *Grin2c*, *Epha10*, *Ptpru*, and *Crtac1*, with downregulation of *Syp*, *Bdnf, Negr1*, and *Gsto1*, each of which has been linked to AD [70–76]. On the other hand, *Ntsr1*, *Kcnh7, Map4k3*, and *Col11a1* are upregulated in *Trem2*^R47H^; 5xFAD over 5xFAD. No consistent effects can be attributed to either the 5xFAD or *Trem2*^R47H^ in subcortical non-thalamic inhibitory and excitatory neurons.

Hippocampal CA1 excitatory neurons (Fig. 7B) exhibited upregulation of the expression of *Ntrk2* and *Mapk1*, associated with the MAPK signaling pathway, in *Trem2*^R47H^; 5xFAD compared with 5xFAD animals. CA3 excitatory neurons showed several differentially expressed genes (*Rph3a*, *Itga7, Hs3st1*), but almost exclusively in the 5xFAD vs. WT comparison, though *Ntsr1* was upregulated in both *Trem2*^R47H^; 5xFAD vs. 5xFAD, and *Trem2*^R47H^ vs. WT comparisons. The dentate gyrus showed upregulation of *Dkk3* and downregulation of *Adgra1* in both 5xFAD dependent comparisons.

Inhibitory cell types (Fig. 7C) consistently exhibited upregulation of *Epha10* in 5xFAD compared with WT, and downregulation in *Trem2*^R47H^; 5xFAD compared with 5xFAD. Few other genes (*Fos, Hrh3)* were consistently differentially expressed between genotypes.

Differentially expressed genes between *Trem2*^R47H^; 5xFAD and WT genotypes (Figs. 6, 7, Supplementary Table 5) matched with the other four comparisons, in that up/downregulation matched that expected by the previous comparisons (e.g. differential expression follows trends in 5xFAD and *Trem2*^R47H^ induced alterations). However, the sensitivity is likely lower, as due to loss of one WT sample, only two imaging batches contained both WT and *Trem2*^R47H^; 5xFAD genotypes. Critically, the results indicating upregulation of *Ntrk2* are replicated in this comparison.

Subclustering subcortical and hippocampal neurons (Supplementary Fig. 6) reveals greater genotype proportion heterogeneity than in cortical excitatory neurons. In contrast with cortical excitatory neurons, hippocampal and thalamic excitatory neurons clustered into large numbers of variable genotype proportion subclusters. Thalamic excitatory neurons subcluster into three genotype enriched sets, including a 5xFAD/*Trem2*^R47H^; 5xFAD enriched subtype a *Trem2*^R47H^/*Trem2*^R47H^; 5xFAD enriched subtype and a WT*/Trem2*^R47H^ enriched subtype. CA1 excitatory neurons identified 6 genotype enriched subclusters, though two of them are spatially localized in the ventral CA1, which is not included in some samples. These include two 5xFAD/*Trem2*^R47H^; 5xFAD enriched subtypes and two WT*/Trem2*^R47H^ enriched subtypes. CA3 excitatory neurons subclustered into 5 genotype biased subclusters, including several localized to the ventral hippocampus (Fig. 7D, ventral hippocampus clusters not shown). One subcluster, labeled C2, present primarily in 5xFAD and *Trem2*^R47H^; 5xFAD samples, is spatially positioned in the intersection of the CA3 and dentate gyrus. This cluster upregulated *Rph3a* and *Dkk3* (Supplementary Fig. 6C).

Overall, neuronal populations exhibit transcriptional alterations associated with both 5xFAD and *Trem2*^R47H^ mutations. In cortical excitatory neurons, these changes are frequently replicated across cell types. Thalamic excitatory neurons, uniquely among subcortical populations, exhibit significant 5xFAD and *Trem2*^R47H^ induced transcriptomic alterations. In the hippocampus, the CA1 shows the most transcriptional alteration among genes measured in this study. Neuronal subclusters show both genotype enrichment, and spatial localization, implying regional population variations induced by 5xFAD and *Trem2*^R47H^ mutations.

## Discussion

*Trem2*^R47H^ is strongly associated with the development of Late-Onset Alzheimer’s Disease. Here, we investigated the spatial transcriptomic impacts of this critical mutation in the context of the 5xFAD mouse model of amyloidosis, as well as in a WT mouse background. Across 19 coronal slices we profiled over 400,000 cells and examined transcriptome dysregulation in neuronal and glial cell types. This analysis provides a broad perspective, enabling analysis of regional and cell type-specific transcriptome dysregulation at the single-cell level. We improved on previous spatial transcriptomic analyses of Alzheimer’s disease mouse models, which were generally limited either in imaged area or in spatial resolution.

To our knowledge, this is the first study to analyze spatial transcriptomics in Alzheimer’s mouse models across whole coronal sections at the single-cell level. Our findings can be organized into four major ideas. First, we identified cell type-specific, plaque-induced differential expression common to both *Trem2*^R47H^; 5xFAD and 5xFAD mouse models. Several of these genes have previously been linked to Alzheimer’s, and in L6b and cortical amygdala excitatory neurons, we identified differentially regulated gene groups specifically associated with learning and memory. Second, we identified unique distribution characteristics for disease associated microglia and astrocytes in relation to plaque density and structural region, with disease associated microglia and astrocytes with high concentrations of microglia in the corpus callosum and subcortical regions, while DAAs were almost exclusively localized to corpus callosum and hippocampus. Third, we identified consistent transcriptomic alterations across cortical, hippocampal, and subcortical neuronal populations, linking cortical excitatory neurons to alterations in BDNF signaling induced by the *Trem2*^R47H^ mutation. Finally, we identified neuronal subtypes enriched and depleted in 5xFAD or *Trem2*^R47H^; 5xFAD that are spatially localized with respect to plaque density. Several of these subtypes show transcriptomic alterations related to learning and memory.

Previous characterization of *Trem2*^R47H^; 5xFAD brains found an initial effect whereby the presence of the variant impeded the microglial response to plaques and exacerbated surrounding dystrophic neurites. However, this suppression of microglial response to plaques subsided at later disease stages, resulting in expected numbers of plaque-associated microglia, and no significant changes in DAM gene expression via bulk-tissue RNA-seq between *Trem2*^R47H^; 5xFAD and 5xFAD hippocampuses [22]. This is consistent with our findings here in regions exhibiting early plaque development (hippocampus, limbic cortex), with hypothesized delay in regions of low plaque development (midbrain, thalamus, hypothalamus) due to later development of plaques in these areas, potentially resulting in a region-specific time-dependent switch in microglia concentration and activation. Despite a seemingly appropriate microglial response to plaques at these later stages, this previous study observed unique emergence of an interferon signature by TREM2^R47H^, coupled with increased plasma neurofilament light chain, a marker of neuronal damage [22]. In agreement with this, we found that the presence of TREM2^R47H^ has significant impacts on gene expression within neurons. As indicated, both microglia and astrocytes showed much higher impacts from the 5xFAD transgenes than from the *Trem2*^R47H^ mutation, resulting in a larger number of differentially expressed genes, and higher magnitude expression changes in both 5xFAD dependent comparisons than in the *Trem2*^R47H^ dependent comparisons. Of those few genes that were differentially expressed in a *Trem2*^R47H^ dependent manner, several are associated with a homeostatic microglial state (*P2ry12*, *Tmem119*), in *Trem2*^R47H^; 5xFAD compared with 5xFAD mice. TREM2 is required to transition microglia from a homeostatic to a DAM state, and the lack of downregulation of these homeostatic markers is consistent with a partial loss of TREM2 ability to mediate this transition with the R47H mutation.

Both disease-associated microglia and astrocytes exhibited distinct spatial distributions, with significant concentrations of both populations in the corpus callosum (CC), though DAM were distributed more evenly across the brain, while DAA were restricted almost exclusively to the CC. While proportions of disease associated astrocytes were consistent between 5xFAD and *Trem2*^R47H^; 5xFAD mouse models, 5xFAD proportions of disease associated microglia were higher than *Trem2*^R47H^; 5xFAD in the dentate gyrus, midbrain, thalamus and hypothalamus, correlating with plaque density. Previous studies have shown indications of neuronal loss in 5xFAD mouse models, particularly in lower cortical layers and the subiculum, though the effect size, statistical significance, and analysis mechanisms vary widely across the literature. Our analysis did not identify any statistically significant neuronal loss, however we do note that the previously identified locations of neuronal loss exhibit remarkably high plaque, DAA and DAM concentrations [77].

Analysis of plaque proximal transcriptomic dysregulation identified the prominent disease associated microglia and astrocyte signatures, with some distance-dependent variations (e.g. *P2ry12* was identified as downregulated near plaques for microglia within 100 µm, but not between cells within 100 µm, and those 100–500 µm from the nearest plaque, with the reverse true for *Tmem119*). *Cd74* was not identified as upregulated near plaques but was strongly upregulated in 5xFAD and *Trem2*^R47H^; 5xFAD mice (compared with WT and *Trem2*^R47H^ respectively). Upregulation of this gene is thought to precede final differentiation into DAM states [78], and exhibits homogeneous upregulation induced by 5xFAD independent of plaque proximity.

Neurons also demonstrated transcriptional alterations commensurate with plaque proximity, which demonstrate little to no overlap with genotype (5xFAD or *Trem2*^R47H^) induced differentially expressed genes. Of those genes upregulated near plaques, many are associated with synaptic transmission including receptors (*Grm1*, *Htr1a, Cnr1*), synaptic vesicle traffic (*Rph3a*), and regulation of neuronal signaling pathways (*Adgra1*). Genes downregulated near plaques are associated with neuronal growth and survival (*Ngf*, *Ntf3*), and synaptic transmission and regulation (*Nptx1*, *Camk2g, Kcnd2*, *Syt6*).

Examining genotype induced differential expression across cortical excitatory cell types, we identified *Fos*, *Wfs1*, and *Grm2* as downregulated by the *Trem2*^R47H^ mutation and *Ntrk2*, *Bdnf*, and Cdh12 as upregulated. Downregulation of *Fos* and *Grm2* is indicative of a decrease in activity and synaptic transmission, and is consistent with the LTP impairments observed in these mice at this age [22]. *Wfs1* is a marker for a unique neuronal population in the entorhinal cortex that modulates spatial memory and is implicated in late stage induced hypoactivity in the hippocampal formation, and is also linked to Tau pathology [67, 79]. *Nr2f2* and *Bdnf* are both critical to the BDNF signaling pathway, which activates ERK and Akt pathways to maintain neuronal survival and synaptic plasticity [80].

Thalamic excitatory neurons exhibited both 5xFAD and *Trem2*^R47H^ impacts, and many differentially expressed genes between WT and 5xFAD mice, including downregulation of *Bdnf*, have previously been linked to Alzheimer’s. CA1 excitatory neurons showed the largest number of differentially expressed genes among non-neocortical neurons, among genes in this panel. However, relatively few differentially expressed genes were identified between 5xFAD and WT mice. The only consistent differentially expressed genes induced by the *Trem2*^R47H^ mutation were *Ntrk2* (upregulated in *Trem2*^R47H^ and *Trem2*^R47H^; 5xFAD) and *Fos*, similar to results in cortical excitatory neurons. Inhibitory neurons exhibited consistent differential expression of *Epha10* (upregulated in 5xFAD compared with WT and downregulated in *Trem2*^R47H^; 5xFAD compared with 5xFAD). This gene is part of the ephrin family and is critically involved in memory formation, with knockout impacts of related receptors (Epha3/4) resulting in reduction in context dependent memory [70].

Most neuron types also exhibit genotype-specific enriched and reduced subclusters, primarily in 5xFAD and *Trem2*^R47H^; 5xFAD mice. Several subclusters exhibit spatial localization, such as the L5 NP subcluster spatially localized near the subiculum, and the L2 IT subcluster spatially localized in the visual and retrosplenial cortices.

Together, our spatial transcriptomic analysis of the effect of the *Trem2*^R47H^ mutation on transcriptional dysregulation across both cortical and subcortical brain regions identified plaque and genotype-dependent transcriptional alterations, cell type-specific transcriptome alterations, and genotype specific cell sub-types spatially localized across the brain.

## Supplementary information

Supplemental Table 1

Supplemental Table 2

Supplemental Table 3

Supplemental Table 4

Supplemental Table 5

Supplemental Table 6

Supplemental Figure 1

Supplemental Figure 2

Supplemental Figure 3

Supplemental Figure 4

Supplemental Figure 5

Supplemental Figure 6

## Data Availability

Raw and processed data are available at the brain image library (/bil/lz/kjohnst2/b72faf9d87d7fc00). We will comply with the NIH and MODEL-AD consortium requirements for data sharing.

## Code availability

Associated Code is available at the brain image library (/bil/lz/kjohnst2/b72faf9d87d7fc00). We will comply with the NIH and MODEL-AD consortium requirements for data sharing.
