# Supplementary material for: Single-cell spatial transcriptomics reveals distinct patterns of dysregulation in non-neuronal and neuronal cells induced by the Trem2R47H Alzheimer’s risk gene mutation
Source: Mol Psychiatry. Author manuscript; Available in PMC 2025 Mar 1. (PMC11746152; doi:10.1038/s41380-024-02651-0)
Supplement: Supplemental Figure 6 [file NIHMS2043213-supplement-Supplemental_Figure_6.pdf]

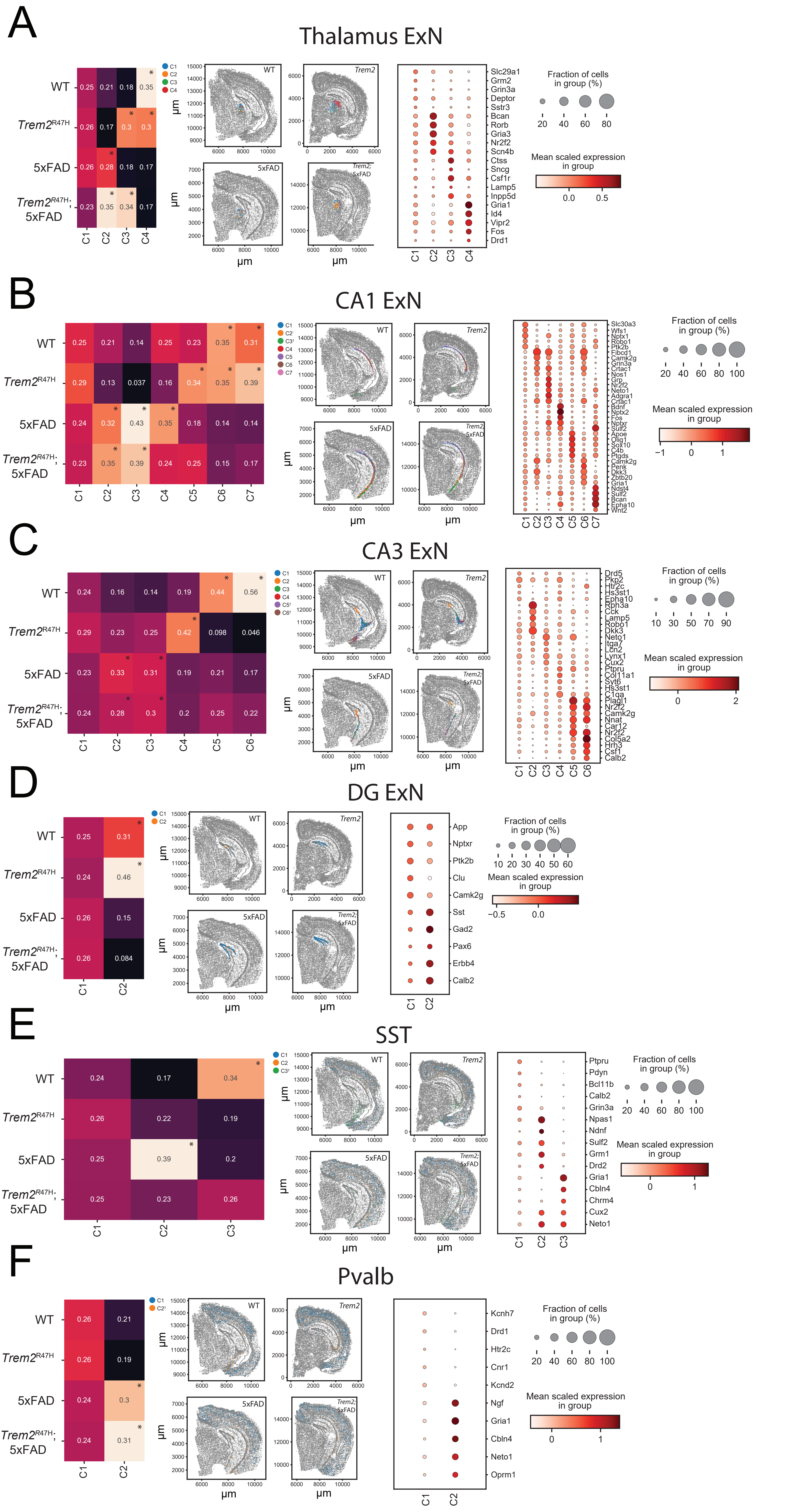

†Spatially biased by sample location

Supplemental Figure 6: Genotype proportions, spatial localization, and subcluster markers for additional subcortical, hippocampal and inhibitory cell types.

A-F: Genotype proportion (left, significant genotype bias indicated with asterisk), spatial localization in 4 samples (one from each genotype, middle), and differentially expressed genes by subgroup (right, dotplot colored by mean scaled expression), for additional neuronal cell types. Daggered clusters indicate those biased toward a specific genotype due to slice location on anterior-posterior axis.
