# Supplementary material for: Single-cell spatial transcriptomics reveals distinct patterns of dysregulation in non-neuronal and neuronal cells induced by the Trem2R47H Alzheimer’s risk gene mutation
Source: Mol Psychiatry. Author manuscript; Available in PMC 2025 Mar 1. (PMC11746152; doi:10.1038/s41380-024-02651-0)
Supplement: Supplemental Figure 3 [file NIHMS2043213-supplement-Supplemental_Figure_3.pdf]

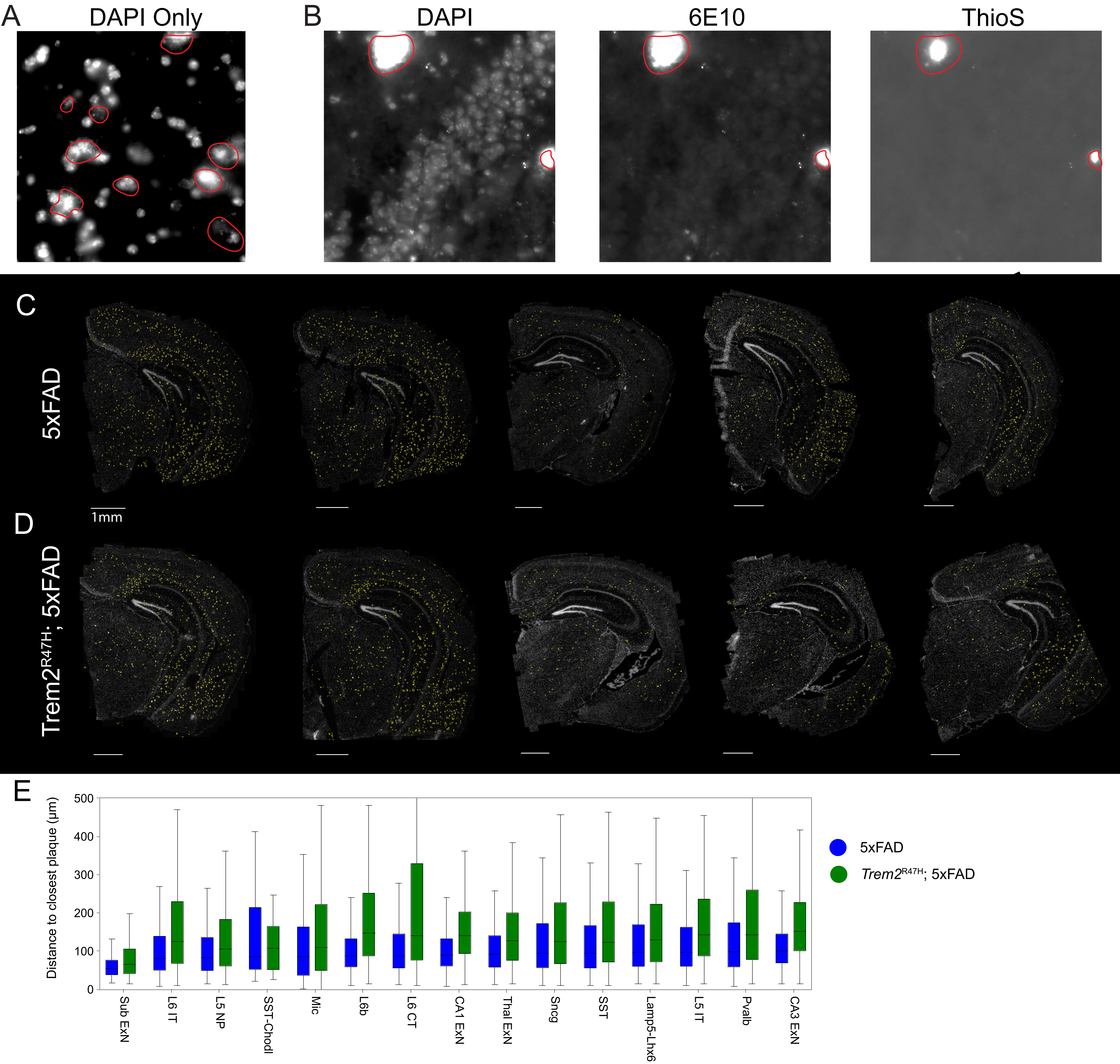

Supplemental Figure 3: Plaque detection and cell type proximity statistics.
A: 2000 x 2000 pixel ROI (0.108 microns per pixel) from DAPI stained 5xFAD section. Red curves indicate boundaries of model predicted plaque boundaries. **B**: 2000 x 2000 pixel ROI from combined DAPI, 6E10 andThioS stained section imaged via MERSCOPE. Red curves indicate model predicted plaque boundaries. Image separated into DAPI (left), 6E10 (middle) and ThioS (right) channels. **C-D**: Detected plaque locations for each 5xFAD (**C**) and *Trem2*^{R47H}; 5xFAD (**D**) sample. **E**: Proximity to closest plaque for the top 15 cell types identified as most plaque proximal (by median distance to plaque).
