# Supplementary material for: Single-cell spatial transcriptomics reveals distinct patterns of dysregulation in non-neuronal and neuronal cells induced by the Trem2R47H Alzheimer’s risk gene mutation
Source: Mol Psychiatry. Author manuscript; Available in PMC 2025 Mar 1. (PMC11746152; doi:10.1038/s41380-024-02651-0)
Supplement: Supplemental Figure 1 [file NIHMS2043213-supplement-Supplemental_Figure_1.pdf]

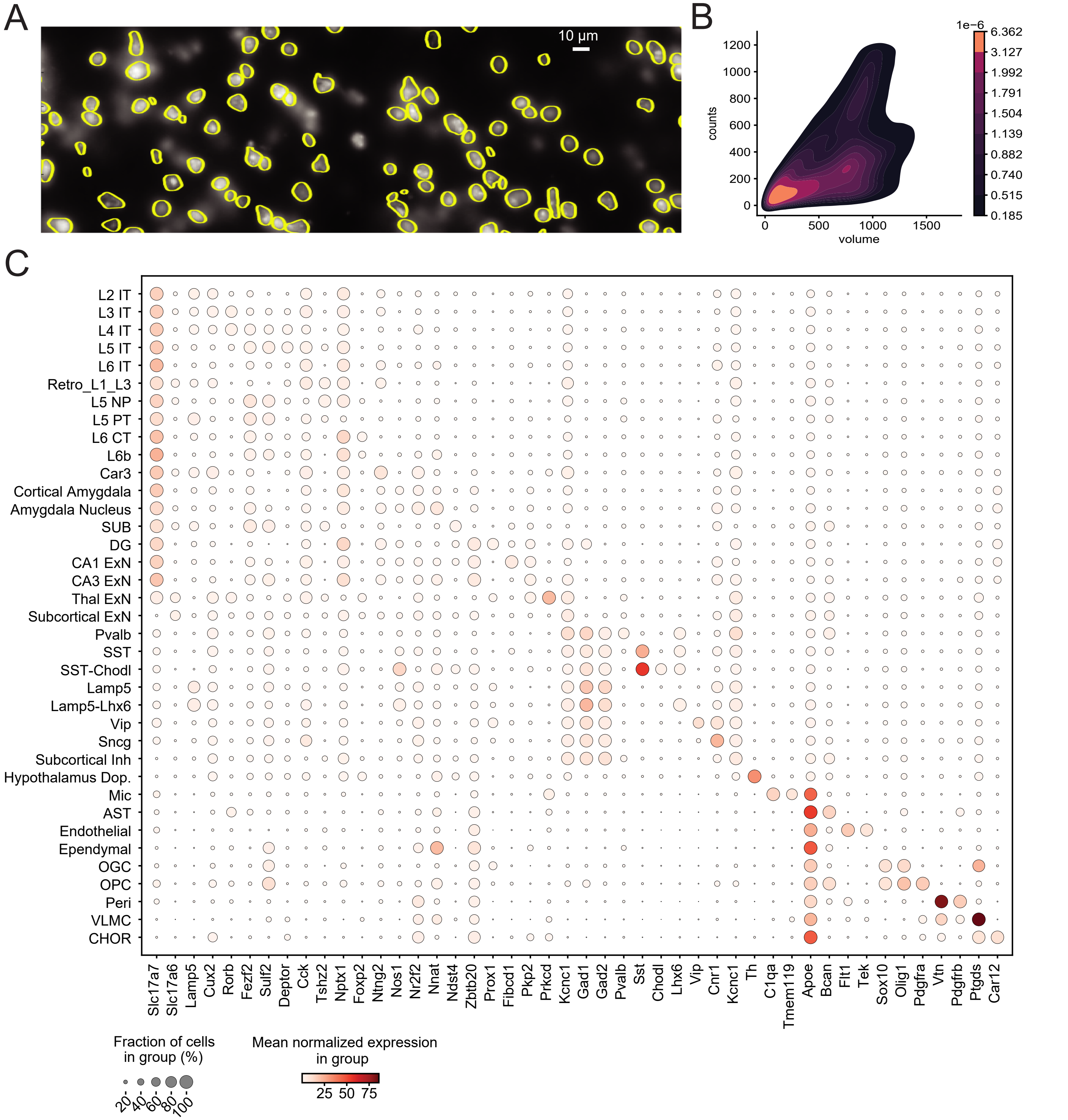

Supplemental Figure 1: Quality control and cell type markers.
A: Sample cellpose segmentation results. **B**: Aggregate density plot indicating counts and volume per cell after quality control. **C**: Dot plot of normalized expression indicating marker genes used to identify individual cell types.
