# Supplementary material for: Single-cell spatial transcriptomics reveals distinct patterns of dysregulation in non-neuronal and neuronal cells induced by the Trem2R47H Alzheimer’s risk gene mutation
Source: Mol Psychiatry. Author manuscript; Available in PMC 2025 Mar 1. (PMC11746152; doi:10.1038/s41380-024-02651-0)
Supplement: Supplemental Figure 2 [file NIHMS2043213-supplement-Supplemental_Figure_2.pdf]

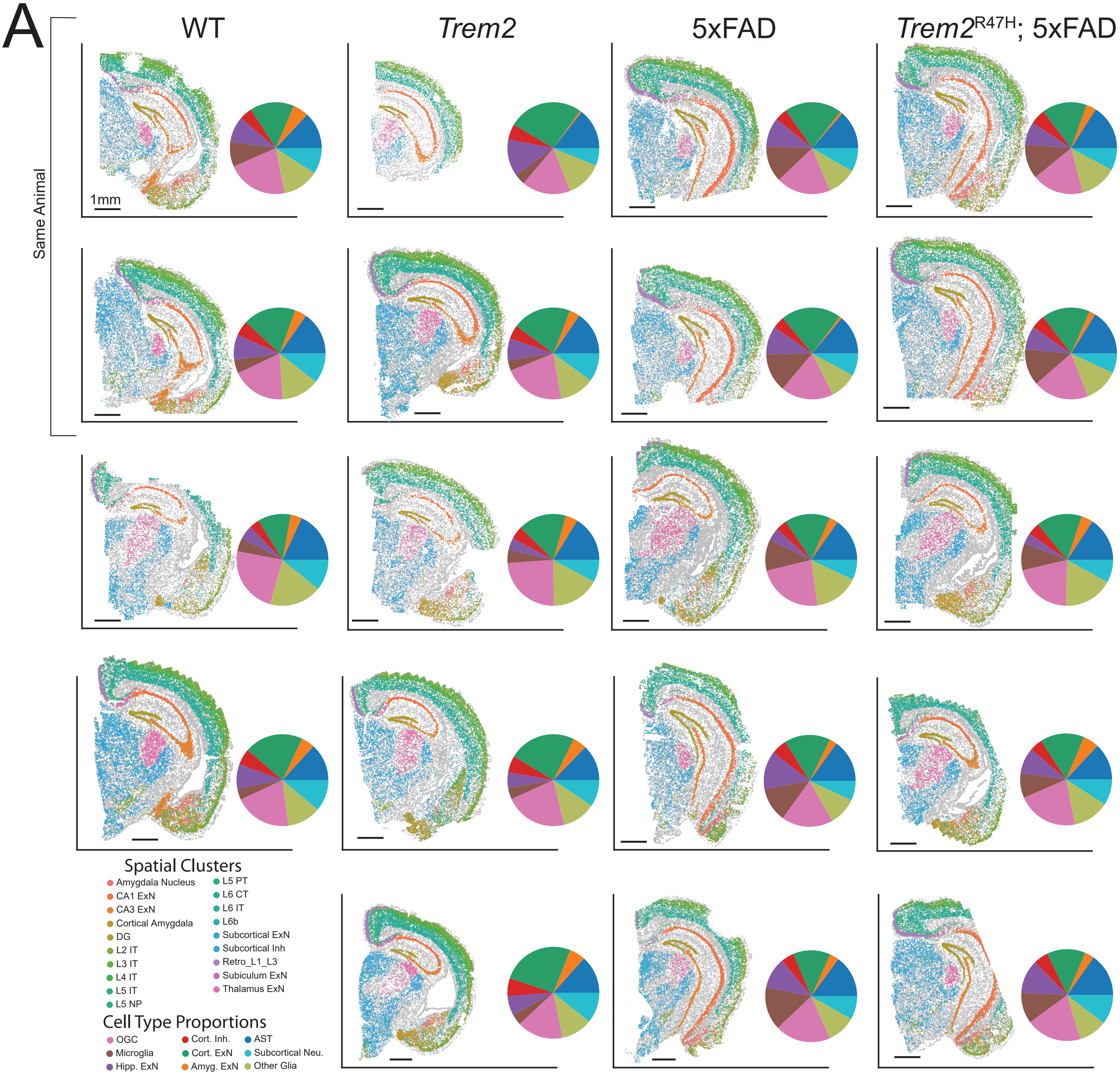

Supplemental Figure 2: Sample overview and cell type proportions.
A: Neuron cell types overlaid in each individual sample in this study, with accompanying cell type proportion pie chart. Legends located in the bottom left of this figure.
