# Supplementary material for: Single-cell spatial transcriptomics reveals distinct patterns of dysregulation in non-neuronal and neuronal cells induced by the Trem2R47H Alzheimer’s risk gene mutation
Source: Mol Psychiatry. Author manuscript; Available in PMC 2025 Mar 1. (PMC11746152; doi:10.1038/s41380-024-02651-0)
Supplement: Supplemental Figure 4 [file NIHMS2043213-supplement-Supplemental_Figure_4.pdf]

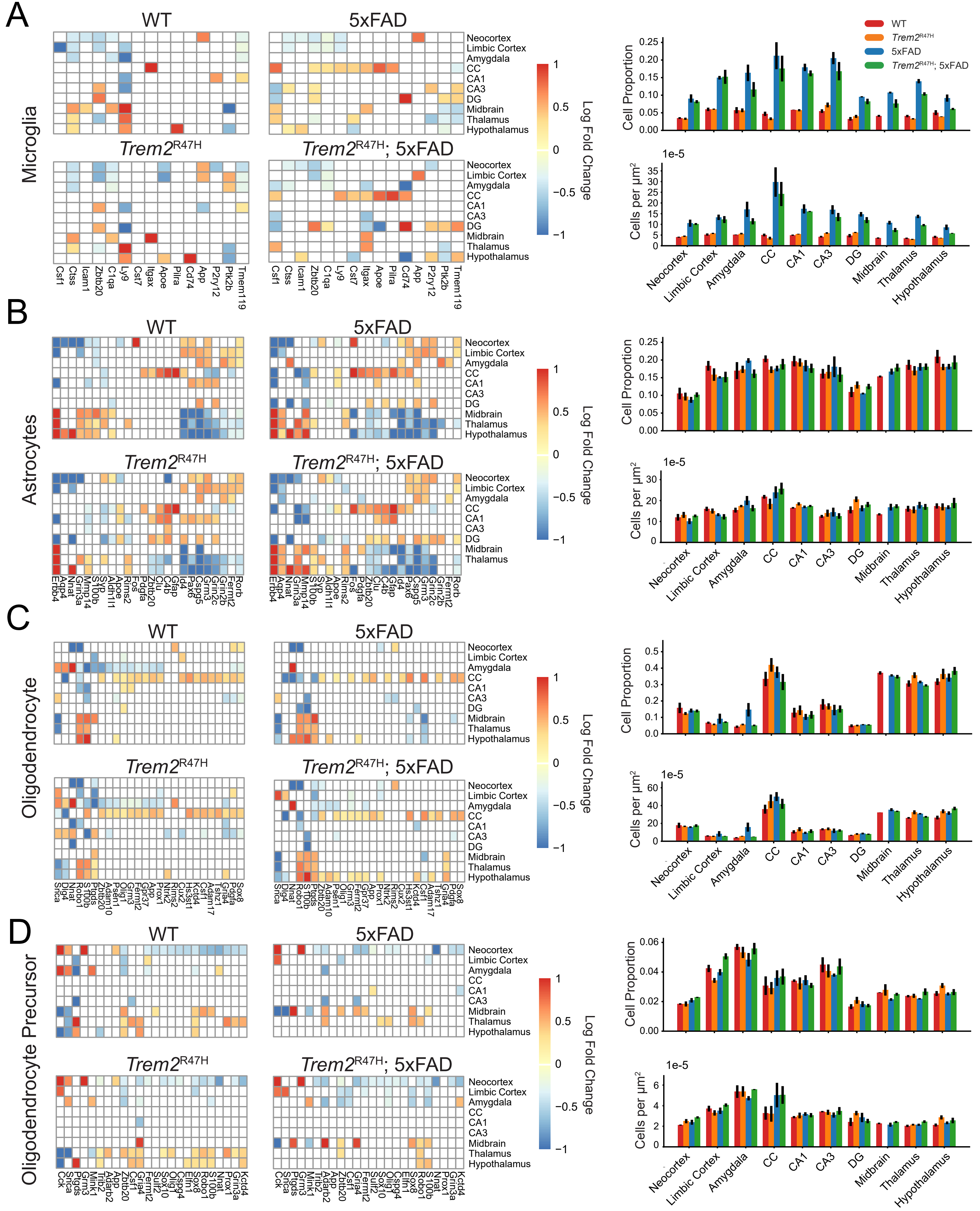

Supplemental Figure 4: Regional transcriptome and cell type proportion variation for major glial cell types.

A-D: Regional transcriptional variations for each genotype (left) combined with cell type proportions and cell density, aggregated by genotype (right). Displayed error bars show standard error (computed across number of samples per genotype). Midbrain data omitted from *Trem2*^{R47H} due to low cell numbers in this region. Microglia (**A**) exhibit relatively little regional variation (left), while both 5xFAD models show increases of up to 200% in microglial proportion and density, across all regions (right). Most (60%) transcriptional variation occurs among disease associated genes. Astrocytes (**B**) exhibit significant regional variations, as was also seen in the subclustering results (**Fig 3B**), largely consistent across genotypes (left), with little variation in proportions or density between genotype in the same region (right). Oligodendrocytes (**C**) exhibit consistent regional variation in Ptgds, Nnat, Robo1, and S100b expression, combined with CC and Amygdala specific gene variation, independent of genotype. Additionally, Robo1 and S100b (among other genes) are also spatially variable in OPC cells (**D**).
